# Prepolymerization Enables On‐Demand Viscosity Tuning for Xolography of Soft Hydrogels

**DOI:** 10.1002/advs.78283

**Published:** 2026-10-26

**Authors:** Xichuan Li, Marcus Reuter, Linguangze Zhuo, Xueheng Dai, Camille Tribout, Nathaniel Corrigan, Jin Zhang, Cyrille Boyer

**Affiliations:** ^1^ Cluster for Advanced Macromolecular Design School of Chemical Engineering University of New South Wales Sydney New South Wales Australia; ^2^ xolo GmbH Berlin Germany; ^3^ School of Mechanical and Manufacturing Engineering University of New South Wales Sydney New South Wales Australia; ^4^ Centre for Advanced Manufacturing Technology (CfAMT) School of Engineering Western Sydney University Sydney New South Wales Australia

**Keywords:** high cyclic compressive durability, multi‐material fabrication, soft hydrogel, support‐free geometry, viscosity adjustment, volumetric additive manufacturing

## Abstract

Xolography enables contactless volumetric additive manufacturing by suspending objects in a viscous resin. This avoids the build platforms and shear forces that distort soft materials in layer‐by‐layer printing. However, low‐viscosity resins such as hydrogels allow printed parts to sediment, and conventional thickeners can compromise the final material. Here, we report an in situ prepolymerization strategy that raises viscosity without altering the base resin composition. A room‐temperature redox reaction converts a fraction of the monomer into high‐molecular‐weight linear chains, tuning viscosity by more than four orders of magnitude (4.37 to >90000 mPa s) through reaction time alone. Across six formulations, longer prepolymerization suppresses sedimentation and lowers the critical light dose by up to 40%, enabling high‐fidelity printing of complex hydrogels within 4 min. The strategy extends to a solvent‐free organic resin, achieving 80 µm resolution. Sequential overprinting yields multi‐material objects with mechanically isolated rigid components embedded in a hydrogel matrix, a geometry difficult to produce by platform‐based printing. The resulting hydrogels retain 99.3% of their peak stress over 5000 compression cycles.

## Graphical abstract

A facile in situ prepolymerization strategy enables on‐demand viscosity tuning across three orders of magnitude without external thickeners, transforming low‐viscosity resins into printable formulations for Xolography. The approach suppresses sedimentation, lowers curing dose, enables high‐fidelity support‐free fabrication, and produces advanced multimaterials with outstanding mechanical resilience.

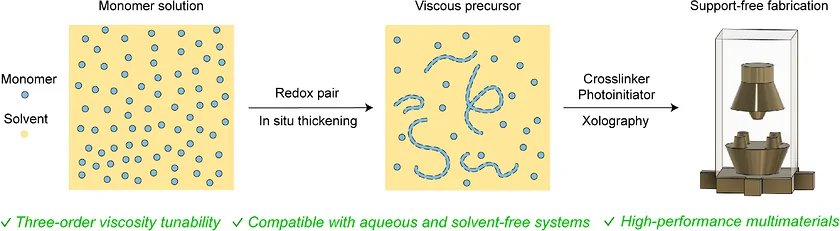

## 1 Introduction

Soft hydrogels occupy a unique position in materials science as their high‐water content, tunable mechanical properties, and biocompatibility make them indispensable for tissue engineering scaffolds [1, 2, 3, 4], soft robotic actuators [5, 6, 7], and flexible electronics [8, 9]. However, fabricating hydrogels into complex three‐dimensional geometries remains a persistent challenge. Conventional vat photopolymerization, encompassing laser scanning stereolithography (SLA) [10], digital light processing (DLP) [11], and continuous liquid interface production (CLIP) [12], builds objects layer by layer from a build platform. The relative motion between the solidifying body and the surrounding liquid resin generates shear and suction forces that potentially impair material quality with layer delamination and breakage [13]. Furthermore, support structures are required to maintain the predesigned shape of soft hydrogels, while adding the complexity of post‐processing removal, accompanied by potential damage to the target hydrogels [14]. More fundamentally, platform‐dependent fabrication restricts designs to fully connected geometries, precluding structures with isolated internal components. Such structures are of considerable interest in biomaterials, such as cardiac valve prostheses, joint replacement models, and encapsulated cell constructs [15, 16].

Volumetric additive manufacturing (VAM) has been developed to circumvent these constraints. Rather than stepwise solidification from a substrate, VAM selectively builds material at arbitrary points within a liquid resin volume, with solidified and uncured regions remaining stationary relative to one another [17]. This contactless fabrication eliminates both the build platform and the need for solid support structures, and, critically for soft materials, removes the shear forces that distort complex geometries during printing. Several techniques have been recently developed. For instance, two‐photon lithography confines polymerization within a highly localized focal volume through nonlinear absorption, achieving resolution below 65 nm [18], with recent scalable platforms exceeding 10^8^ voxels s^−1^ [19]. Computed axial lithography (CAL), or tomography, reconstructs three‐dimensional objects by projecting sequential two‐dimensional light patterns into a rotating resin container, enabling fabrication of centimeter‐scale objects in seconds [20], with recent improvements by rotating light patterns to achieve sub‐second high‐fidelity fabrication [21]. Among these, Xolography offers a distinctive combination of speed, resolution, and multi‐material capability without requiring iterative hologram optimization or specialized equipment beyond a dual‐color light source [22].

Xolography achieves contactless fabrication through a dual‐color photoinitiator (DCPI) that sequentially absorbs two wavelengths of light to generate free radicals only at the intersection of a 405 nm laser sheet and visible‐light images (referred to as red light) projected by a digital light processor [23, 24, 25, 26, 27]. Because solidification requires both wavelengths simultaneously, regions exposed to only one, or neither, remain liquid. This mechanism enables continuous fabrication of centimeter‐scale objects with low surface roughness within minutes, and has been demonstrated across an expanding range of geometries and materials, including flow cells [22], waterwheels [23], overprinted multi‐material structures [28], and hydrogel constructs [29, 30]. For soft hydrogel applications in particular, Xolography can fabricate isolated internal shapes, a geometry class inaccessible to platform‐based methods [16].

Central to all VAM approaches, however, is resin viscosity. In contactless fabrication, sufficient viscosity is essential to prevent relative motion between solidified and uncured regions: without it, gravity causes printed parts to sink or drift within the surrounding liquid before the resin has been fully processed, irreversibly distorting the geometry. For Xolography, resins with viscosity above approximately 1000 mPa s are typically required to attain support‐free structures [23, 28, 31]. This presents a fundamental formulation challenge for hydrogel systems, which are inherently low‐viscosity solutions containing a significant amount of water. Two strategies have been pursued to overcome this problem. Low gravity, specifically, microgravity conditions generated during a parabolic flight campaign, enabled precise Xolography printing of resins as low as 11 mPa s, demonstrating that the viscosity requirement is physical rather than chemical in origin [32]. While this approach requires specialized operational conditions and operates within specific 20‐s fabrication windows, it serves as a valuable proof of concept. Consequently, resin optimization is more widely adopted for standard workflows. Reactive macromonomers can be incorporated to formulate inherently viscous resins [23, 29], while non‐reactive rheological modifiers, such as nanoparticles, offer an alternative route to adjust viscosity [31]. Implementing high solid‐content nanoparticles requires careful calibration, as it can influence optical transparency and light scattering, which affect print fidelity [33]. To manage this, refractive‐index‐matching agents can be introduced to balance the optical properties, though they necessitate adjustments to the overall formulation [16, 34, 35]. Ultimately, because the compatibility of a given thickener depends on the specific resin system, identifying the ideal additive combination involves a tailored, system‐specific development process.

In adhesive manufacturing, multi‐stage polymerization strategies have been widely utilized in the production of pressure‐sensitive adhesives and tapes [36, 37, 38]. In the initial stage, ultraviolet irradiation is used to generate photocurable viscous syrups with viscosities ranging from 300 to 20 000 mPa s, which facilitate uniform coating onto appropriate substrates. Subsequently, a second curing stage, sometimes involving the addition of crosslinking agents, is employed to further increase monomer conversion and complete network formation [36]. Inspired by this development in the field of coating, we propose the introduction of a non‐photoinitiated prepolymerization (or dark polymerization) strategy that tunes resin viscosity without altering the base chemical resin composition in additive volumetric manufacturing. Prior to printing, a room‐temperature redox reaction converts a small fraction of the monomer into high‐molecular‐weight linear chains, yielding viscous resins that serve as printing precursors. Precursor viscosity is highly tunable across more than four orders of magnitude simply by varying the prepolymerization time. Because only a minor fraction of the monomer is consumed during this initial prepolymerization reaction, the remainder remains fully available for subsequent photopolymerization during Xolography printing. At the optimal oxidizer concentrations, the targeted precursor viscosity is attained via efficient quenching and remains stable over 14 days of storage. We demonstrate that this prepolymerization‐mediated viscosity tuning broadens the printable light‐dose window, reduces the critical solidification energy by up to 40%, and suppresses gravity‐induced sedimentation over extended print durations, thereby enabling the high‐fidelity fabrication of exceptionally soft hydrogels containing more than 90 wt.% water. As a proof of concept, we successfully extend this method to solvent‐free, low‐viscosity organic resins, achieving high‐resolution printing (80 µm) and the fabrication of a complex functional waterwheel. Furthermore, we leverage this approach to print multi‐materials via Xolography, fabricating a mechanically functional articular joint model that comprises two mechanically isolated rigid components seamlessly embedded within a soft hydrogel matrix. Beyond broadening the processing window for Xolography, the in situ generated high‐molecular‐weight polymers do not compromise the mechanical performance of the resulting hydrogels. Long‐term cyclic compression testing reveals excellent elastic recovery and mechanical stability with negligible fatigue. Ultimately, this in situ thickening strategy requires no additional chemical components, providing a design principle for adapting low‐viscosity resin systems to contactless volumetric fabrication. Because the viscosifier is generated in situ from the resin's own monomer and requires no modification to the established dual‐color workflow, the strategy should be transferable to other free‐radical resin chemistries. This work successfully extends contactless volumetric fabrication to both aqueous and organic low‐viscosity systems, thereby broadening material design opportunities and enhancing manufacturing versatility.

## 2 Results and Discussion

### 2.1 Operating Principle of Xolography

Xolography achieves support‐free fabrication by combining a dual‐color photoinitiator (DCPI) with two perpendicular light sources (Figure 1a). A violet laser sheet (405 nm, ≈50 µm thick) is formed by two counter‐propagating beams that widen symmetrically to compensate for attenuation through the resin [22]. Upon irradiation with violet light, the spiropyran moiety of DCPI undergoes ring opening to generate a metastable merocyanine intermediate, which thermally reverts to the inactive spiropyran form in the dark (Figure 2b). On‐demand 2D images projected by a digital light processor in the visible‐light range (520 & 613 nm) then activate the merocyanine, which abstracts a hydrogen atom from a tertiary amine coinitiator to generate radicals and initiate polymerization [23]. Because free‐radical generation requires absorption of both wavelengths, polymerization is strictly confined to their intersection. As the violet sheet advances relative to the resin with synchronously updated red‐light images, centimeter‐scale complex objects are fabricated continuously within minutes and with low surface roughness [39, 40]. Print resolution is governed by the total light dose delivered to the resin, which is set by two independently adjustable parameters: vat translation speed and violet (405 nm) light‐sheet intensity (Equation S1). Reducing translation speed or increasing light intensity raises the energy dose, promoting faster polymerization and higher crosslink density.

**FIGURE 1:**
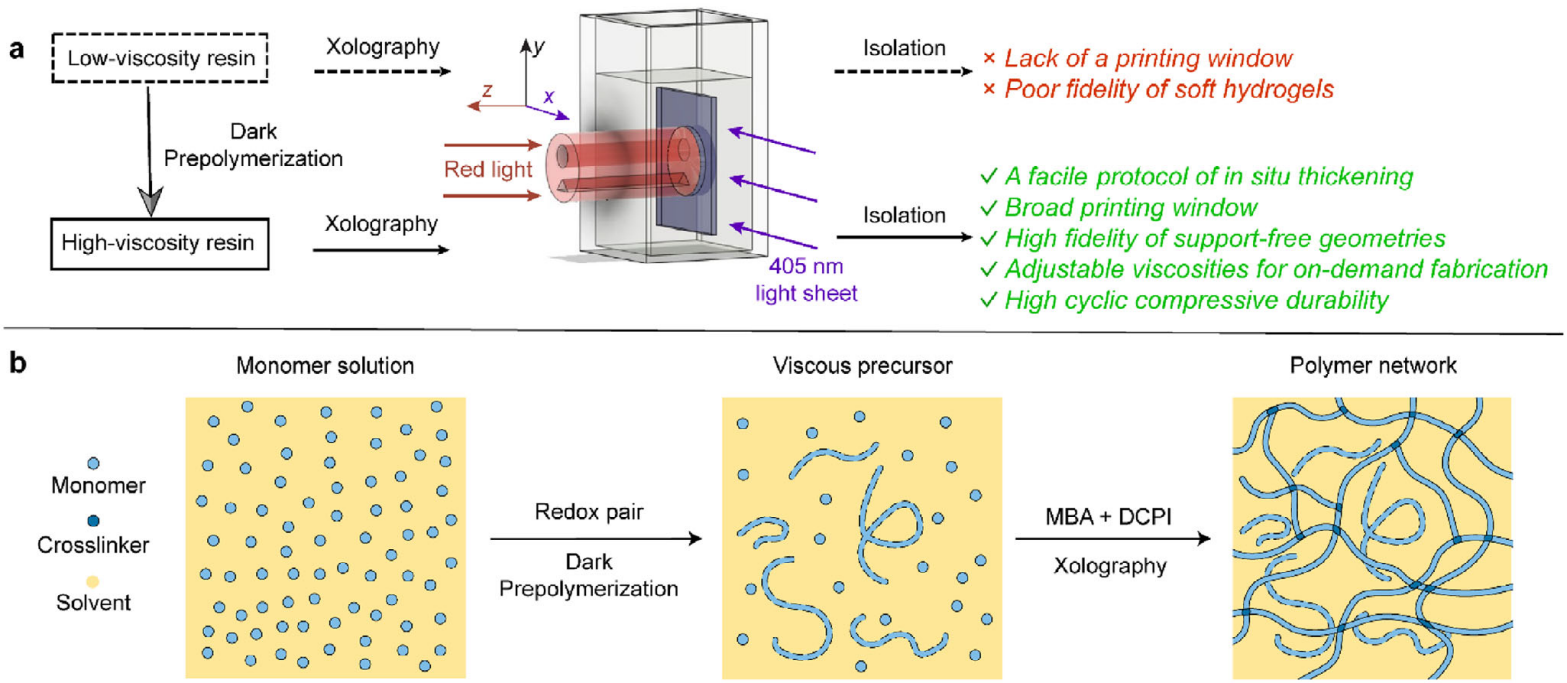
Xolography of low‐viscosity aqueous resins enabled by dark prepolymerization. (a) Schematic of the Xolography process: a 405‐nm violet laser sheet and synchronously projected visible‐light images (520 & 613 nm) intersect within the resin volume to confine polymerization to their overlap region; printability comparison between the unmodified low‐viscosity resin (dashed line) and the prepolymerized resin (solid line) across a range of applied light doses. (b) Chemical scheme illustrating in situ viscosity enhancement of an aqueous hydrogel resin via dark redox prepolymerization, yielding a viscous precursor, followed by addition of crosslinker (MBA) and photoinitiator (DCPI) to give a print‐ready resin for Xolography.

**FIGURE 2:**
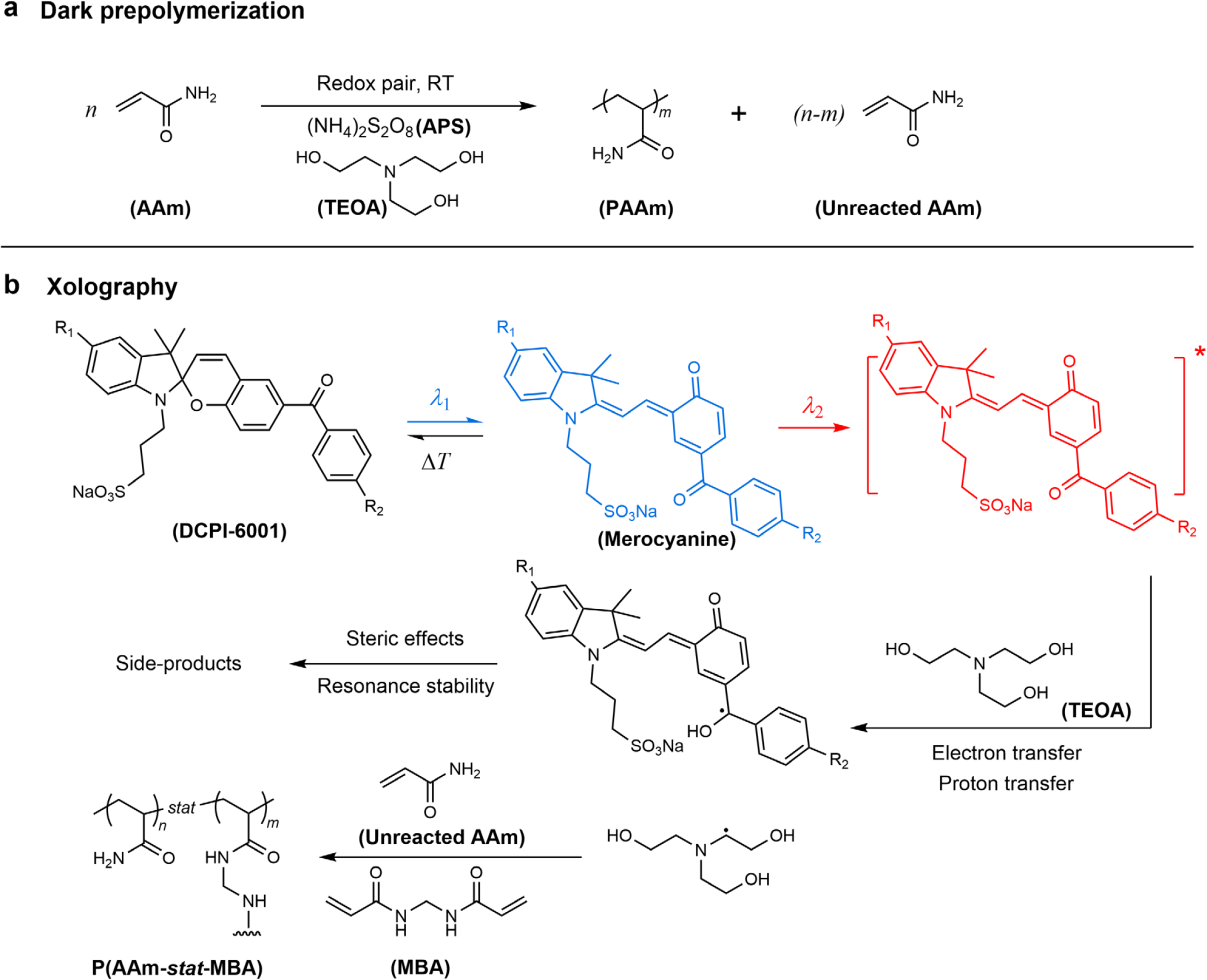
Mechanism of dark prepolymerization and subsequent Xolography. (a) Redox‐initiated prepolymerization of acrylamide (AAm) by an ammonium persulfate/triethanolamine (APS/TEOA) redox pair at room temperature (RT, 24 °C), yielding a viscous precursor comprising high‐molecular‐weight polyacrylamide (PAAm) chains and unreacted AAm monomer available for subsequent photopolymerization. (b) Dual‐wavelength photoinitiation mechanism of the water‐soluble DCPI‐6001: sequential absorption of 405‐nm and red‐light photons generates free radicals in the presence of a tertiary amine coinitiator, confining polymerization to the beam intersection and enabling high‐fidelity Xolography of the viscous precursors prepared in (a).

Beyond the light‐dose parameters discussed above, printable resins for Xolography must satisfy three criteria. First, the resin must be optically transparent and fully homogeneous: absorbing components or undissolved species that attenuate or scatter the 405 nm light as it propagates through the vat can result in an inhomogeneous intensity profile that degrades spatial resolution and print fidelity. This effect becomes more severe with increasing build depth. Second, the resin must support rapid liquid‐to‐solid transitions upon irradiation; sluggish polymerization kinetics allow partially solidified features to sediment or drift before the fabrication is complete, distorting the final geometry. Third, and central to this work, the resin must possess sufficient viscosity, typically above 1000 mPa s [23, 28, 31], to suppress convective motion between solidified and uncured regions during contactless fabrication. Generally, the sinking rate is inversely proportional to the viscosity [32]. It is this last requirement that distinguishes Xolography resins from those used in conventional vat photopolymerization, and that motivates the viscosity‐tuning strategy developed in this work [41].

### 2.2 Optimization of Experimental Procedures and Resin Formulation

In this work, we developed an approach for the synthesis of hydrogels, which are typically challenging to print via VAM methods without using viscosity modifiers due to their inherently low viscosity [42]. As a model hydrogel resin, we selected acrylamide (AAm) and *N, N*′‐methylenebisacrylamide (MBA) as the monomer and crosslinker, respectively, which were dissolved in deionized (DI) water. Such formulations are widely used to prepare hydrogels for drug delivery [43], batteries [44], and strain sensors [45]. A water‐soluble dual‐color photoinitiator (DCPI‐6001) and triethanolamine (TEOA) as a co‐initiator were incorporated to enable Type II photoinitiation and render the system compatible with Xolography (Figure 2b). As anticipated, however, this base resin proved unprintable across the full range of applied light doses, which is due to its inherently low viscosity and sluggish polymerization kinetics. While this issue could theoretically be addressed by adding pre‐synthesized, high‐molecular‐weight polymers directly into the formulation, this approach requires the polymer to be sourced or synthesized separately and is practically hindered by the notoriously slow and inefficient dissolution kinetics of high‐molecular‐weight polymers (see the direct comparison in Section 2.4). To overcome this bottleneck, viscosity was modulated in situ prior to printing through a dark redox prepolymerization process, using ammonium persulfate (APS) and TEOA as the oxidant/reductant pair to partially polymerize the AAm monomer. Two protocols were evaluated: a one‐step process, in which all resin components were present during prepolymerization, and a two‐step process, in which the crosslinker was introduced only after prepolymerization was complete.

#### 2.2.1 One‐Step Protocol

In the one‐step protocol, all resin components, including monomer (AAm, 45 wt.%), crosslinker (MBA, 0.04 wt.%), DI water (47.4 wt.%), comonomer (DEAm, 2.5 wt.%), DCPI (0.05 wt.%), TEOA (5 wt.%), and APS (0.0006 wt.%), were combined in a single pot and subjected to prepolymerization prior to Xolography (Figure S1). For this approach to be viable, the resin must become sufficiently viscous through the formation of long linear polymer chains while avoiding premature crosslinking and gelation. MBA concentrations were gradually reduced from 0.5 to 0.04 wt.%; however, even the formulation with 0.06 wt.% MBA underwent gelation before reaching the targeted viscous yet flowable state (Figure S1a). Consequently, 0.04 wt.% MBA was selected for subsequent printing experiments, as this concentration enabled the formation of a viscous and processable precursor. This behavior is consistent with classical gelation theory for vinyl/divinyl copolymerization. Because redox prepolymerization generates very long primary chains (M_n_ ≈ 160–225 kg mol^−1^, Đ ≈ 2.2 for the crosslinker‐free precursors; Table 1), approximately one crosslinked unit per weight‐average primary chain is sufficient to reach the gel point. Flory–Stockmayer estimates [46, 47], assuming equal reactivity of all vinyl groups, place the ideal gel point at approximately 12–17% and 18–26% monomer conversion for 0.06 and 0.04 wt.% MBA, respectively (depending on primary‐chain length), i.e., within the conversion range required to obtain printable viscosities (15–28% for H3–H6; Table 1). Although intramolecular cyclization delays gelation in practice, these estimates explain why only trace crosslinker levels are compatible with the one‐step protocol. Prior to prepolymerization, the resin mixture was degassed to remove dissolved oxygen. Subsequently, the resin was stirred at room temperature to allow the redox polymerization to proceed. The reaction was then quenched by exposing the mixture to atmospheric oxygen (air) and immediately placing it into an ice bath while stirring. This quenching procedure was common to both the one‐step and two‐step protocols.

**TABLE 1:** Characterization of thickening precursors and resins as a function of prepolymerization time.

ID	Prepolymerization time (s)	Precursor				Resin^d^
		Monomer Conversion^a^ (%)	*M*_n_ ^b^ (kg mol^−1^)	*Ð* ^b^	Viscosity^c^ (mPa s)	Viscosity^c^ (mPa s)
**H1**	0	0	N/A	N/A	4.37	3.10
**H2**	590	5.90 ± 0.10	223.9 ± 4.2	2.23 ± 0.04	145.7 ± 7.7^*^	73.36 ± 1.41
**H3**	815	15.15 ± 0.38	201.4 ± 4.0	2.22 ± 0.02	5172 ± 207	1539 ± 57
**H4**	950	20.55 ± 0.14	195.0 ± 1.2	2.20 ± 0.02	21376 ± 1091^*^	5246 ± 224
**H5**	1070	24.52 ± 0.06	191.5 ± 2.1	2.17 ± 0.01	51720 ± 1182	11037 ± 412
**H6**	1110	28.35 ± 0.34	161.4 ± 11.3^*^	2.12 ± 0.03	94447 ± 4909^*^	18597 ± 1397^*^

^a^ Monomer conversion was determined by ^1^H NMR from the integral ratio of CH_2_ and CH protons of monomers and prepolymers, with D_2_O as solvent.

^b^ Number‐average molecular weight (*M*_n_) and polymer molecular weight distribution (*Ð*) were characterized by aqueous SEC (phosphate buffer (pH = 9) as the eluent, poly(ethylene oxide) as the standards).

^c^ Dynamic viscosity was measured at 25 ± 0.2 °C using a viscometer equipped with a CPA‐52Z spindle for H2–H6 and a CPA‐42Z spindle for H1. The viscosity of H1‐H6 precursors was measured at 384, 40, 3, 0.4, 0.2, 0.1 s^−1^, respectively. The viscosity of H1‐H6 resins was measured at 384, 100, 10, 2.0, 1, 0.4 s^−1^, respectively.

^d^ Resins were prepared by adding crosslinker (MBA) and DCPI‐6001 to the prepolymerized precursors. Data for H2–H6 are presented as mean ± SD (*n* = 3; population SD). Relative standard deviation (RSD) was calculated based on the equation: $\mathrm{RSD}\;=\frac{\mathrm{SD}}{\mathrm{mean}}\times 100\%$. 5% ≤ RSD < 10% for asterisked data, and RSD < 5% for other data.

At the optimized light dose and a fixed print speed of 1 mm min^−1^, well‐defined disk‐shaped objects were fabricated via Xolography. However, isolating intact structures without compromising their fine features proved challenging due to the low crosslink density imposed by 0.04 wt.% MBA formulation, which resulted in weak hydrogels with poor handling properties (Figure S1b). Attempts to improve mechanical robustness by increasing the MBA concentration to 0.06 wt.% caused premature gelation during prepolymerization, rendering the precursor resin unprintable (Figure S1a‐iii). Consequently, the one‐step protocol was unable to simultaneously satisfy the competing requirements of high precursor viscosity and adequate final crosslink density, motivating the development of the two‐step approach described below.

#### 2.2.2 Two‐Step Protocol

Given the limitations of the one‐step approach, a two‐step protocol was developed to decouple viscosity development from crosslink density, allowing both to be optimized independently. In this protocol, AAm (45 wt.%), TEOA (5 wt.%), APS (0.0006 wt.%), and deionized water were first subjected to redox prepolymerization at room temperature (24 °C) for a controlled duration to reach a specific target viscosity. Subsequently, the crosslinker solution (MBA in DI water, 3 wt.%) and photoinitiator solution (DCPI in DEAm, 2 wt.%) were sequentially added to the resin precursor to produce a print‐ready formulation, resulting in final concentrations of 0.5 wt.% MBA and 0.05 wt.% DCPI (Table S1). Introducing the crosslinker only after the prepolymerization stage eliminated the risk of premature gelation. This strategy enabled a significantly higher MBA content relative to the one‐step protocol (0.5 wt.% vs. 0.04 wt.%), successfully ensuring excellent structural integrity and mechanical robustness in the final printed hydrogel.

The DCPI concentration was fixed at 0.05 wt.% based on optical transparency constraints. UV–vis spectroscopy confirmed strong absorption at 405 nm attributable to DCPI (Figure S2a), and a calibration series determined a molar extinction coefficient of *ε* = 544 L mol^−1^ cm^−1^ at this wavelength (Figures S2b,c). Light distribution modelling across three DCPI concentrations (0.01, 0.05, and 0.25 wt.%) demonstrated the spatial profile when two counter‐propagating laser beams irradiated the resin from opposite sides. Due to the absorption of DCPI, the light‐sheet intensity was lowest at the center of the vat and highest near the walls [23]. Increasing DCPI concentration from 0.01 to 0.25 wt.% significantly aggravated light attenuation, causing the intensity drop between the edge and the center to surge from 0.5% to 82.2% (Figure S2d). This extreme attenuation results in severe underpolymerization at the center of the cuvette at elevated DCPI loadings. Consequently, while higher initiator concentrations accelerate photoinitiation kinetics, 0.05 wt.% was selected as the optimal balance to ensure both sufficient initiation efficiency and adequate light penetration depth.

The APS concentration was also optimized to establish a highly controllable viscosity‐tuning window within a short prepolymerization period while avoiding the Trommsdorff–Norrish effect (Table S2). Because the polymerization rate scales with the square root of the initiator concentration [48], lower APS loadings reduce the polymerization kinetics, thereby affording finer control over the final viscosity endpoint. At the highest loading tested (0.0016 wt.%), residual APS continued the polymerization after exposure to air. This was accompanied by a pronounced auto‐heating profile characteristic of the Trommsdorff–Norrish (gel) effect, rendering precise viscosity control impractical. Reducing the concentration to 0.0008 wt.% eliminated these thermal and kinetic effects, while maintaining an acceptable reaction time (392 s). A further reduction to 0.0006 wt.% broadened the accessible viscosity range without excessive prolongation of the reaction (590 s), whereas 0.0004 wt.% extended prepolymerization to 892 s with no meaningful improvement in controllability. Consequently, an APS loading of 0.0006 wt.% was therefore selected for all subsequent experiments.

The optimized formulation thus comprises AAm (45 wt.%), MBA (0.5 wt.%), TEOA (5 wt.%), APS (0.0006 wt.%), DCPI (0.05 wt.%), and deionized water (46.95 wt.%), with 2.5 wt.% *N, N*‐diethylacrylamide (DEAm) included to aid DCPI dissolution in the aqueous precursor resin (Table S1).

### 2.3 Viscosity Control via Prepolymerization

With the optimized formulation and procedures, prepolymerization time was used as the sole variable to tune monomer conversion and, consequently, resin viscosity. All precursors were prepared within 30 min by redox prepolymerization at room temperature by varying the reaction time between 0 and 1110 s. The resulting precursor viscosities ranged from 4.37 mPa s (H1, no prepolymerization) to 94447 ± 4909 mPa s (H6). Across H2–H6, the precursor viscosity scaled approximately as a power law of PAAm concentration, with an exponent of ≈ 4.7 over 3.3–15.8 wt.% PAAm (Table 1), consistent with the scaling of entangled semidilute polymer solutions (as viscosities were measured at different shear rates, this exponent is approximate). The precursors therefore lie above the critical entanglement concentration from the early stage of prepolymerization (H2–H3), which accounts for the viscosity increase of several orders of magnitude at low monomer conversion [49]. In this regime, entanglements and non‐covalent interactions dominate the rheological response, producing large viscosity gains without reaching the gel point. In addition, prepolymerization kinetics followed a quasi‐first‐order trend after an induction period [50], while auto‐acceleration above ≈ 25% monomer conversion (the apparent first‐order rate constant increased from ≈ 4.6 × 10^−^^4^ s^−^^1^ for H2–H5 to ≈ 1.3 × 10^−^^3^ s^−^^1^ between H5 and H6) reduced viscosity controllability (Figure S3c). Prepolymerization was therefore limited to ≤ 28% monomer conversion (H6), where the endpoint remains controllable by reaction time. The corresponding resin, after addition of DCPI and MBA, displayed a viscosity from 3.10 to 18597 ± 1397 mPa s (Table 1).

During prepolymerization, AAm monomers were progressively consumed to form linear PAAm chains, as confirmed by ^1^H NMR spectroscopy, which showed monomer conversion increasing monotonically with reaction time (Table 1 and Figure S3a). SEC characterization revealed high‐molecular‐weight PAAm chains with broad, initially bimodal molar mass distributions that narrowed toward unimodal with increasing conversion (Figure S3b). This evolution reflects the decrease in kinetic chain length as the monomer is consumed [50, 51], the longest chains are formed early, when the monomer concentration is highest, whereas chains initiated at higher conversion are shorter, so that *M*_n_ decreases with reaction time despite the concurrent rise in overall polymer content. Chain transfer to TEOA, whose concentration remains constant while the monomer is depleted, may also contribute. Importantly, the unreacted monomer fraction retained after prepolymerization remains fully available for photopolymerization upon addition of MBA and DCPI, allowing the final printed hydrogel to maintain sufficient crosslink density and mechanical integrity (Table S5).

Following the quenching protocol (i.e., exposure to atmospheric oxygen and immediate placement in an ice bath with stirring), the precursor resin exhibited excellent stability. Only a negligible increase in monomer conversion was detected after 24 h of storage on rotating rollers. This confirms that the combined thermal and oxidative quenching method reliably suppresses ambient dark polymerization, preserving precursor properties for extended storage windows (Table S3). For the most viscous precursor (H6), monomer conversion remained almost unchanged after 14 days of storage (Figure S12c‐ii). Batch‐to‐batch reproducibility was validated across three independent replicates per formulation (H2–H6), where each batch was prepared and polymerized for the identical target duration. These viscosity measurements consistently exhibited a relative standard deviation below 5%, with the sole exceptions marked by asterisks still falling securely below 10%, demonstrating that a specific target viscosity can be delivered on demand. Such tightly controlled reproducibility fulfills a critical requirement for any viscosity‐tuning strategy intended for routine additive manufacturing workflows (Table S4).

### 2.4 Viscosity‐Tunable Resins for Support‐Free Xolography

Having established viscosity control via prepolymerization, we turned our attention to the printability of the five resulting resins (H2–H6). These were formulated by adding MBA solution and DCPI solution to the respective prepolymerized precursors, yielding final viscosities from 73.36 to 18597 mPa s (Table S5). An unprepolymerized control resin (H1, 3.10 mPa s) was included across all experiments to confirm that the low‐viscosity base formulation was unprintable by Xolography.

Printability was assessed using three geometrically distinct models of increasing fabrication complexity: a thin dumbbell with a high aspect ratio, a round disk with two types of hollow features, and a multi‐pointed star with sharp tips (Figures 3 and 4). The high‐aspect‐ratio dumbbell model was selected due to its exceptional sensitivity to gravity‐induced distortion during printing and its mechanical fragility during subsequent isolation, making it uniquely suited for mapping the printable light‐dose window of each formulation. The disk and star models introduce enclosed hollow cavities and fine, high‐curvature details, respectively, providing a rigorous test of spatial resolution and geometric fidelity. All three models were fabricated at a fixed translation speed of 1 mm min^−1^ across part thicknesses of 1, 2, and 4 mm, allowing the impact of precursor viscosity on print quality to be systematically evaluated as a function of extended fabrication time.

**FIGURE 3:**
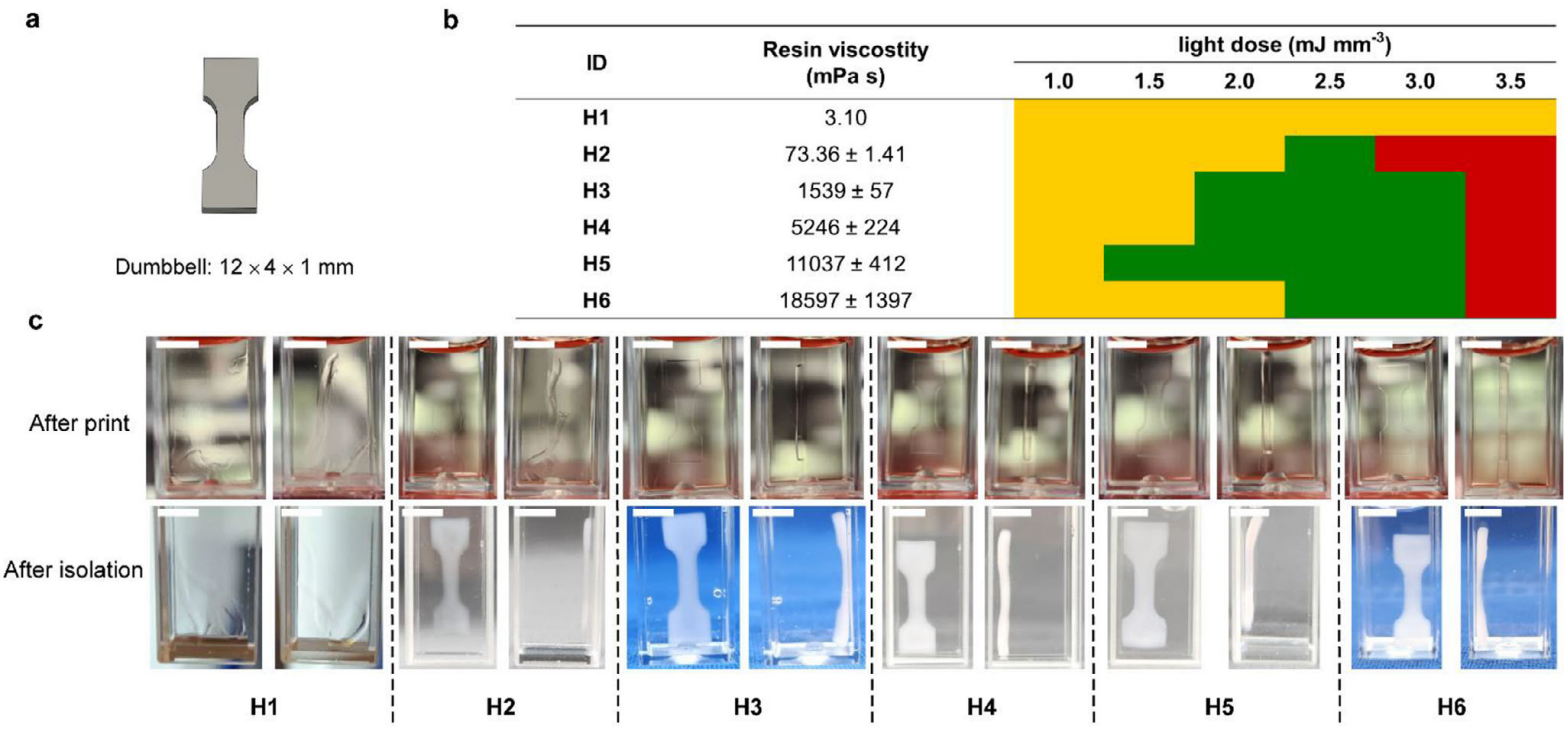
Light‐dose printability windows for hydrogels fabricated via the prepolymerization protocol (H1–H6). (a) dumbbell model used to define the printability window for each formulation; the high aspect ratio and 1 mm thickness make this geometry sensitive to gravitational distortion during printing and mechanical failure during isolation. (b) Light‐dose‐based printability windows at a fixed translation speed of 1 mm min^−1^, categorized by outcome after isolation: under‐cured (yellow), well‐cured (green), and over‐cured (red); the width of the green band indicates the accessible fabrication window for each resin. (c) Representative photographs of bar‐shaped hydrogels printed at the optimized light dose for each formulation, before and after isolation. Scale bars: 5 mm.

**FIGURE 4:**
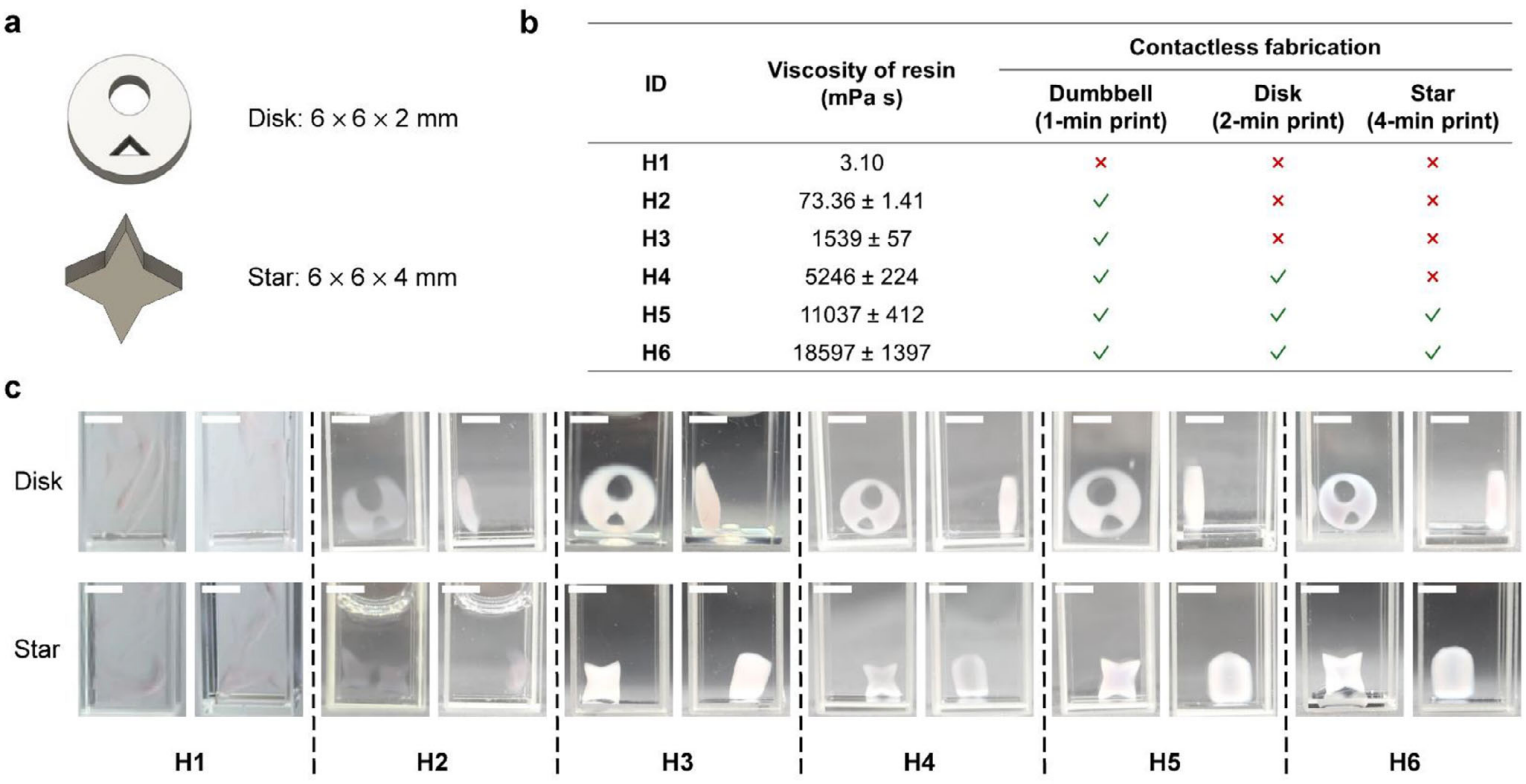
Effect of resin viscosity on geometric fidelity and sedimentation resistance over extended fabrication times. (a) Digital models used for resolution characterization: disk (positive and negative features) and star (sharp tips), each formulation fabricated at three thicknesses (1, 2, and 4 mm) corresponding to fabrication times of 1, 2, and 4 min at 1 mm min^−1^. (b) Printability summary across H2–H6 assessed by two criteria: geometric fidelity along both light‐propagation axes, and gravity‐induced tilting as a judgement of sedimentation resistance during fabrication; color coding indicates outcome at each thickness for each formulation. (c) Representative photographs of disk‐ and star‐shaped hydrogels fabricated at the optimized light dose for each formulation after isolation and washing. Scale bars: 5 mm.

The printability window was mapped using the high‐aspect‐ratio dumbbell model at a fixed translation speed of 1 mm min^−1^, with light dose varied by adjusting the violet light‐sheet intensity (Equation S1). This approach isolates light dose as the sole experimental variable while maintaining a fabrication time short enough to minimize gravitational distortion in lower‐viscosity formulations. Print outcomes were classified into three curing states, namely under‐cured, well‐cured, and over‐cured, and were assessed after isolation and washing rather than immediately after printing (Figure 3b). Under‐curing occurs when the delivered dose is insufficient to drive the resin past the gel point, yielding fragile or shapeless structures. Over‐curing arises when the violet light dose exceeds the upper printability threshold: at high intensities, the 405 nm sheet alone generates sufficient DCPI‐derived radicals to initiate polymerization without red‐light activation, and this single‐wavelength solidification first manifests at the vat walls where light attenuation is lowest (Figure S2d) [22, 23]. Evaluating resolution after isolation rather than immediately after printing is essential due to the low refractive index contrast between the hydrogel and the surrounding liquid resin. It conceals structural defects that only become apparent once the liquid phase is removed, a discrepancy discussed in detail below. Isolated materials placed in water serve as the definitive criterion for evaluating the printability window of each formulation (Figure 3c).

We first examined the printability window as a function of light dose (1.0 to 3.5 mJ mm^−3^) across the full formulation series (H1–H6; Table S5). As anticipated, the unprepolymerized control group (H1, 3.10 mPa s) was unprintable across the entire dose range: gravitational sedimentation and floating of partially solidified material prevented retention of any geometric features, and all H1 prints were classified as under‐cured regardless of applied intensity (Figure S4). The prepolymerized formulations (H2–H6) are therefore the subject of the printability analysis that follows.

With prepolymerization, H2 (73.36 ± 1.41 mPa s) became printable, albeit with a narrow light‐dose window of 2.5–2.75 mJ mm^−3^ (Figure S5): below this range only partial geometries formed, while above it single‐wavelength over‐curing occurred under 405‐nm irradiation. Despite its low viscosity, H2 produced well‐defined shapes with smooth surfaces within this window, attributed to the short fabrication time (1 min at 1 mm min^−1^ for a 1 mm‐thick model). The short fabrication time limits sedimentation, with insignificant gravitational distortion. Dimensional fidelity was confirmed along both the 405‐nm laser‐sheet and red‐light propagation axes, demonstrating that the printable window, though narrow, is sufficient to produce geometrically accurate structures at this thickness.

H3 (1539 ± 57 mPa s) exhibited a broader printable window of 2.0–3.0 mJ mm^−3^ (Figure 3 and Figure S6). This enhanced printability is driven by the elevated viscosity, which suppresses radical termination reactions via the Trommsdorff–Norrish effect while leaving chain propagation largely unaffected, thereby accelerating the overall free‐radical polymerization kinetics [23, 52, 53]. Consequently, a lower critical light dose is sufficient to drive the resin past its gel point. Moreover, because the initial viscosity varies directly with monomer conversion across this formulation series, the more viscous resins inherently possess a higher fraction of pre‐formed polymer chains and thus begin the printing process closer to the gel point. Notably, an H3 print (1 mm min^−1^, 3.5 mJ mm^−3^) that appeared well‐cured before isolation was reclassified as over‐cured after the surrounding liquid resin (whose refractive index closely matches that of the solid hydrogel) was removed, revealing structural defects concealed by the index‐matching effect before post‐curing (Figure S6).

The printable window of H4 (5246 ± 224 mPa s; Figure S7) was comparable to that of H3, suggesting that the incremental increase in viscosity between these two formulations produced only a modest additional acceleration of polymerization kinetics. H5 (11037 ± 412 mPa s) yielded the largest printable window of the series, spanning from 1.5 to 3.0 mJ mm^−3^ (Figure S8). The highest‐viscosity resin, H6 (18597 ± 1397 mPa s), did not extend this window further (Figure S9). Although intact bar‐shaped prints were obtained at 2.0 mJ mm^−3^ before isolation, the resin viscosity was sufficiently high that vigorous washing was required to remove the viscous surrounding medium. This washing process caused mechanical failure of less crosslinked structures (or under‐cured materials), thereby raising the effective minimum well‐cured dose to 2.5 mJ mm^−3^. This demonstrates that printability windows must be defined from post‐isolation assessment, and that beyond optimal viscosity, the effective minimum light dose is set by post‐processing demands rather than solely by photopolymerization kinetics.

Taken together, these results demonstrate that prepolymerization‐driven viscosity tuning has a direct and monotonic effect on the printable light‐dose window up to H5, with the critical minimum dose decreasing from 2.5 mJ mm^−3^ (H2) to 1.5 mJ mm^−3^ (H5), a 40% reduction that tracks the increase in prepolymerization time, and hence in both viscosity and monomer conversion. Beyond H5, excessive viscosity narrows the printability window due to the post‐processing constraint described above. The following section examines how these formulations perform under the more demanding condition of extended fabrication time, where gravitational sedimentation becomes the dominant challenge.

In addition to the dumbbell model, two more complex models, disk and star geometries, were designed to evaluate geometric fidelity and sedimentation resistance over extended print times of 2 and 4 min, respectively (Figure 4). Sedimentation was quantified rather than assessed visually: for each formulation, the settling displacement of the first printed layer relative to the last was measured and converted into a settling velocity and a dimensionless sedimentation number (Equations S7–S9 and Table S6), which decreased systematically with increasing prepolymerization time. Related dimensionless and numerical treatments of part sedimentation in volumetric additive manufacturing have been reported previously [54, 55].

At the optimized light dose for each formulation, resolution was assessed along both the 405‐nm and red‐light propagation axes before and after isolation (Figure S10 and Figure 4c). H2 failed to produce printable disk or star geometries at either thickness, consistent with its marginal viscosity and slow polymerization kinetics. H3 showed improved shape integrity but exhibited significant gravity‐induced tilting along the red‐light axis, indicating that its viscosity was insufficient to resist sedimentation over the longer fabrication time required by these models. H4 supported high‐fidelity printing of the disk model, but visible gravity‐induced tilting was observed in star geometry. As expected, H5 and H6 produced both geometries with high dimensional accuracy on the 405‐nm plane and level surfaces along the red‐light axis (Figure 4c). The sharpness of star‐shaped hydrogels printed with H5 and H6 was further quantified by staining to enhance feature contrast, revealing a minimum resolved feature size of 30 µm (Figure S11). Quantitative measurements of positive, negative, and sharp features across all formulations are compiled in Table S7. The reported dimensions reflect the swollen state of the hydrogels with high water content (Table S8), which is inherent to polyacrylamide networks with low crosslinking density, and is relevant to end‐use conditions such as cell culture scaffolds and soft tissue models demanded in aqueous conditions.

Across the full formulation series, H5 (11037 ± 412 mPa s) emerged as the optimal formulation: it combined the widest printable light‐dose window (1.5–3.0 mJ mm^−3^, Figure 3b) with the ability to sustain high geometric fidelity over 4‐min fabrication time (Figure 4b), and its moderate viscosity allowed straightforward isolation by gentle washing. H6 (18597 ± 1397 mPa s) matched H5 in geometric fidelity for both disk and star models and demonstrated superior sedimentation resistance, an advantage exploited in the multi‐material fabrication described in Section 2.6. However, its narrower printable window (2.5–3.0 mJ mm^−3^), together with the vigorous washing required for isolation, makes it a less versatile choice for single‐material printing.

It is noteworthy that the incorporation of additional formulation components into the prepolymerized precursor may become challenging at extremely high viscosities, such as that of H6 (94447 ± 4909 mPa s). In this formulation, the water content was relatively high (46.95 wt.%; Table S1). A portion of the water was used to dissolve the monomer (AAm) during prepolymerization, while the remainder was employed to prepare the crosslinker solution (3 wt.% MBA). Following the preparation of the H6 precursor, the addition of the MBA solution acted as a diluent, significantly reducing the viscosity and thereby facilitating the incorporation of DCPI, which had been pre‐dissolved in a small amount of DEAm (Table 1). Furthermore, the viscous resins were homogenized using rotating rollers to minimize concentration gradients and ensure uniform formulation composition. For the highest‐viscosity formulation (H6), this mixing process required approximately 2 h to achieve satisfactory homogeneity. This process helped prevent heterogeneity‐induced curing defects, enabling the fabrication of high‐fidelity structures (Figure 4) and mechanically robust hydrogels without observable defects (Figure 6).

Furthermore, a conventional thickening approach based on the addition of an external polymer additive (PAAm) was investigated and compared with the proposed in situ prepolymerization strategy. To ensure a like‐for‐like comparison, PAAm was isolated from the H6 precursor through dialysis and lyophilization, rather than sourced commercially, and subsequently characterized by ^1^H NMR and SEC (Figure S12a). The composition of the H6 precursor and its thickening efficiency were used as the basis for comparison with the conventional thickening method (Figure S12b‐i). Initially, all resin components were mixed in the absence of PAAm, after which PAAm was added in three equal portions, each corresponding to 5.27 wt.% (Figure S12b‐ii). The first PAAm addition was almost completely dissolved after 5 h of stirring at 40 °C, resulting in an increase in viscosity from 4.06 to 1637 mPa s. This viscosity, obtained with one‐third of the target PAAm content, lies between those of the H2 and H3 precursors (145.7 and 5172 mPa s); after dilution with the MBA and DCPI solutions, the corresponding resin would fall below H3 (1539 mPa s), which already showed gravity‐induced tilting in 2–4 min prints (Figure 4b). However, the incorporation of the second PAAm portion proved considerably more challenging, and complete dissolution could not be achieved even after 16 h of alternating heating and roller mixing. The viscosities of the optimal H5 and H6 resins were therefore not accessible by this route. In contrast, the proposed in situ prepolymerization strategy generated highly viscous precursors within less than 30 min, reaching 94447 ± 4909 mPa s for H6 (Table 1; 96 000 mPa s for the batch shown in Figure S12b‐i). Including the ≈ 2 h of roller mixing after addition of the MBA and DCPI solutions, a print‐ready H6 resin was obtained in ≈ 2.5 h, whereas the conventional route had not reached the target composition after more than 21 h. Furthermore, the H6 precursor exhibited higher optical transmittance, reaching 91% at both 405 and 585 nm, compared with the conventionally thickened resin, which showed transmittances of 78% at 405 nm and 83% at 585 nm (Figure S12c‐i). Furthermore, after two weeks of storage, the H6 precursor maintained its optical transparency, with no observable phase separation or precipitation of PAAm. Likewise, the monomer conversion exhibited a negligible increase from 28.4% to 28.9% during storage, and the viscosity increased by less than 7% (96000 vs 102300 mPa s), comparable to the batch‐to‐batch variation (Table 1), demonstrating good storage stability (Figure S12c‐ii). These results highlight the superior efficiency and practicality of the in situ approach for preparing highly viscous resins suitable for Xolographic printing.

### 2.5 Prepolymerization for Solvent‐Free Xolography

To further demonstrate the wide adaptability of this in situ thickening method, a solvent‐free organic system was investigated with bulk prepolymerization and Xolography. Here, *N, N*‐diethylacrylamide (DEAm) and trimethylolpropane triacrylate (TMPTA) were selected as the monomer and crosslinker, respectively, while TEOA and benzoyl peroxide (BPO) were introduced as a redox pair for prepolymerization at room temperature (Figure S13).

As previously described, the BPO concentration was rapidly optimized to obtain a relatively fast protocol of preparing viscous precursors that were still stable after overnight storage on the rotating rollers (Table S9). Briefly, high concentrations of oxidizer (≥ 0.025 wt.%) led to a significant viscosity increase in the precursors after the prepolymerization was stopped, while too low a concentration of BPO (≤ 0.010 wt.%) largely extended the time (> 2 h) to reach the targeted viscosity (> 1000 mPa s). By optimization, 0.015 wt.% BPO balanced a rapid thickening procedure (35 min) with effective viscosity controllability (2322 mPa s for precursor).

Two solvent‐free acrylamide formulations, P1 and P2, were prepared to compare the printability of resins without and with in situ thickening (Table S10). As shown in Figure 5a, the base formulation P1 had a very low resin viscosity (4.12 mPa s). In contrast, dark prepolymerization yielded a viscous P2 precursor within 35 min at room temperature, with a defined monomer conversion and prepolymer content (Figure 5a and Figure S14).

**FIGURE 5:**
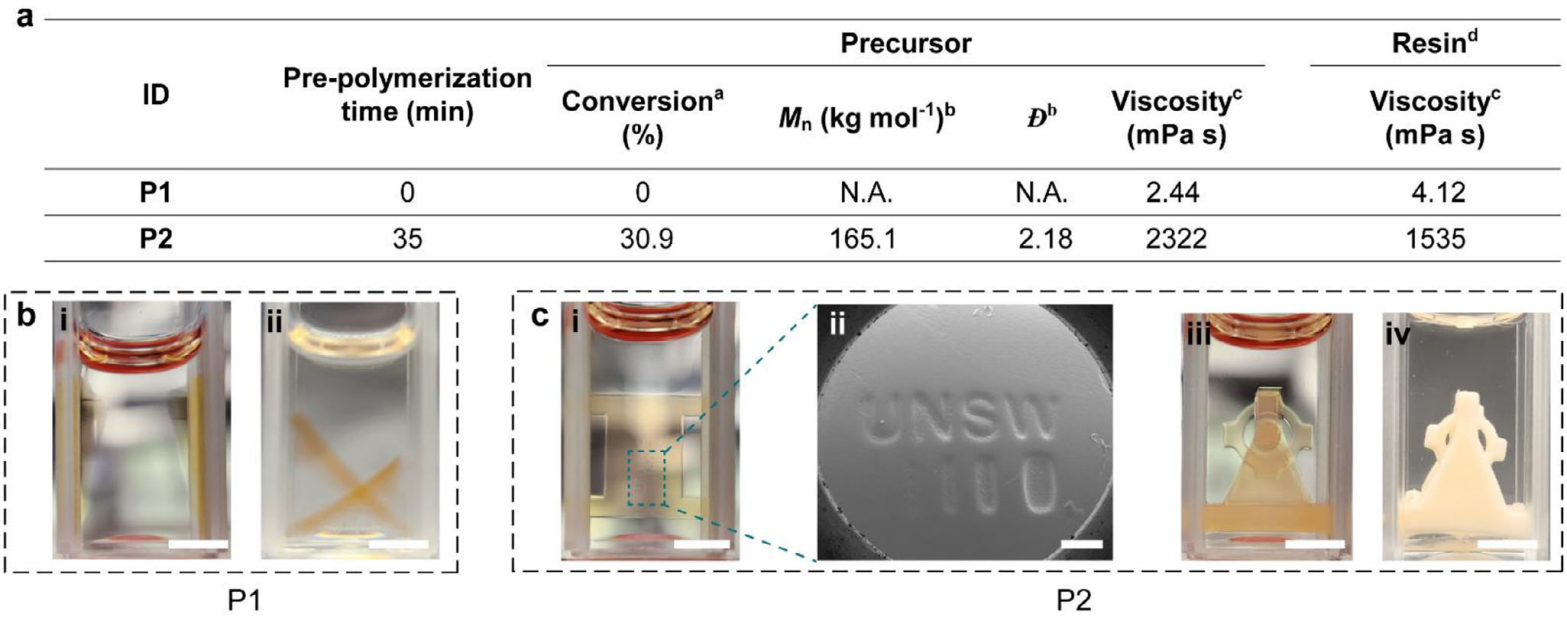
Prepolymerization‐enabled Xolography of a low‐viscosity solvent‐free system. (a) Characterization of thickening precursors and resins as a function of prepolymerization time. ^a^‐Monomer conversion was determined by ^1^H NMR in D_2_O. ^b^‐Number‐average molecular weight (*M*_n_) and polymer molecular weight distribution (*Ð*) were characterized by SEC (DMAc as the eluent, PMMA as the standards). ^c^‐Dynamic viscosity was measured at 25 ± 0.2 °C using a viscometer equipped with a CPA‐52Z spindle for P2 and a CPA‐42Z spindle for P1. ^d^‐Resins were prepared by adding crosslinker (TMPTA) and DCPI‐3001 to the prepolymerized precursor. (b) Photographs of the printed resolution cube (**i**) before and (**ii**) after isolation using P1 formulation, printed at 1 mm min^−1^, 9.0 mJ mm^−3^. (**c**) Xolography of the printed resolution cube showing (**i, ii**) fine features fabricated at 1 mm min^−1^, 1.5 mJ mm^−3^, with five line widths designed to be 320, 160, 80, 40, 20 µm from right to left; and (**iii, iv**) complex geometry of a functional waterwheel printed at 4 mm min^−1^, 1.6 mJ mm^−3^, shown (**iii**) before and (**iv**) after isolation. Scale bars: 500 µm in (**cii)** and 5 mm in all other images.

Both formulations were then evaluated in Xolography using DCPI‐3001 as the photoinitiator, selected for its adaptability to the non‐aqueous resin. After each formulation was combined with the crosslinker TMPTA and DCPI‐3001, the resulting resins were printed from the same resolution cube model. The P1‐based resin yielded only under‐cured material, even at an ultra‐high light dose (9.0 mJ mm^−3^; Figure 5b), whereas the P2‐based resin printed successfully at a far lower dose (1.5 mJ mm^−3^), resolving fine features down to 80 µm (Figure 5c‐i,ii). To illustrate the capability of contactless VAM to fabricate unconnected and interlocked structures, a functional waterwheel with freely moving parts was produced in a single print at 4 mm min^−1^ (11.87 mm^3^ s^−1^), directly and without supports (Figure 5c‐iii,iv and Video S1).

### 2.6 Viscosity‐Tunable Resins for Multi‐Material Xolography

Having established the single‐material printability of H2–H6, we next exploited the contactless nature of Xolography to fabricate multi‐material rigid–soft composite structures by sequential overprinting. This specific capability is fundamentally difficult to replicate by platform‐based methods, such as SLA and DLP printing techniques, where shear forces and support structure requirements preclude the fabrication of fully encapsulated or mechanically isolated components. For multi‐material fabrication, the translation speed of hydrogels increased from 1 to 2 mm min^−1^, raising the volumetric generation rate to 5.93 mm^3^ s^−1^.

To illustrate the overprinting capability, we designed an object containing soft and rigid components, in this case a leaf model (Figure 6a‐i). The rigid component was fabricated from prepolymerized solvent‐free resins (P2), selected for its high crosslink density and mechanical stiffness relative to the high‐water‐content hydrogel (Figure 6a‐ii). The H5 formulation (11037 ± 412 mPa s) was selected for the leaf blade, as its viscosity supported high‐fidelity fabrication with easy recovery as the optimal hydrogel formulation (Figure 6a‐iii). After isolation, the rigid stem was conformally encapsulated within the soft hydrogel lamina with no delamination at the rigid–soft interface (Figure 6a‐iv).

**FIGURE 6:**
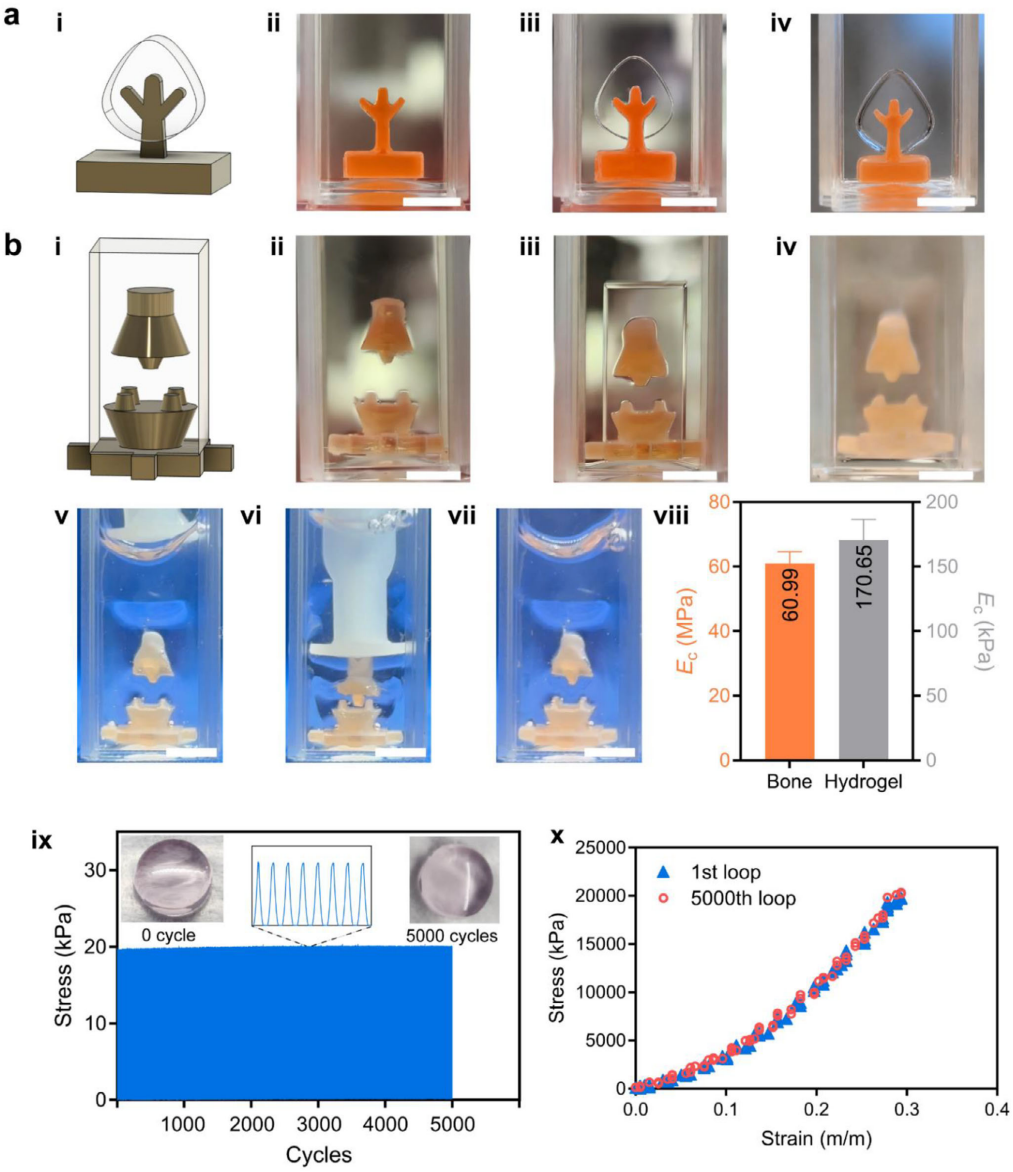
Multi‐material Xolography of rigid–soft composite structures by sequential overprinting. (a) Leaf model fabricated by overprinting: (**i**) target model, (**ii**) rigid stem sitting in the hydrogel resin printed with the prepolymerized solvent‐free P2 formulation, (**iii**) soft leaf blade printed with H5 resin (11037 ± 412 mPa s), and (**iv**) final isolated part showing conformal encapsulation of the rigid stem within the hydrogel blade. (b) Articular joint model fabricated by overprinting: (**i**) target model, (**ii**) two mechanically isolated bone components sitting in the hydrogel resin printed from PETA resin, (**iii**) surrounding soft matrix overprinted in H6 resin (18597 ± 1397 mPa s), the high viscosity of which mitigates bone sedimentation during fabrication, and **(iv)** final isolated assembly. (**v‐vii**) Sequential photos from cyclic compression performance, showing reversible coupling and separation of the joint during loading and unloading, and qualitatively reproducing the engagement and release of an articular joint under cyclic load. (**viii**) Compressive modulus (*E*_c_) of the rigid bone and soft hydrogel (H6). Data are reported as mean ± SD; *n* = 4. (**ix**) Cyclic compression test of a printed disk (H6) at a compression strain amplitude of 30% in aqueous conditions. (**x**) Hysteresis loop curves of the hydrogel at the beginning (1st cycle) and the end (5000th cycle) of cyclic compression testing. All soft hydrogels were fabricated at a translation speed of 2 mm min^−1^. Scale bars: 5 mm.

A geometrically more demanding model, a load‐bearing articular joint comprising two mechanically isolated bone components (rigid materials) embedded in a continuous soft tissue matrix, was then fabricated to demonstrate the full capability of our approach (Figure 6b‐i). In contrast to the soft hydrogels used in this work (*E*_c_ = 170.65 kPa), pentaerythritol tetraacrylate (PETA) was selected to fabricate rigid components, exhibiting a compression modulus (*E*_c_ = 60.99 MPa) more than two orders of magnitude higher than that of the hydrogel (Figure 6b‐viii and Figure S15). The two rigid components were first printed and precisely positioned within H6 resin (18597 ± 1397 mPa s) (Figure 6b‐ii). The highest‐viscosity formulation was selected specifically to mitigate sedimentation of the upper bone component during the 3.5‐min overprinting step, when the surrounding hydrogel matrix was fabricated (Figure 6b‐iii). After isolation and transfer to DI water, the assembled joint was subjected to cyclic top‐down compression as a proof‐of‐concept test of the coupled rigid–soft assembly, conceptually analogous to the reversible loading of an articular joint (noting that the moduli and applied stresses here are not matched to physiological values) (Figure 6b‐v–vii). The soft hydrogel exhibited fully reversible deformation upon load application and release across three compression cycles, confirming the mechanical integrity of the rigid–soft interface and the elastic recovery of the hydrogel matrix (Video S2).

To further investigate cyclic durability, a quantitative compression test was conducted on the pre‐equilibrated hydrogel (H6) in DI water, at a 30% compression amplitude of strain (Figure S16). Over 100 cycles, the stress amplitude remained nearly constant with highly reproducible loading responses (Figure S16a), and the minuscule hysteresis loop enclosed during each loading–unloading cycle confirms negligible energy dissipation with no deterioration of structural integrity through 100 cycles (Figure S16b). Extended testing over 5000 loading–unloading cycles further demonstrated outstanding cyclic compressive durability, with negligible stress attenuation and no visible defects in the gel throughout (Figure 6b‐ix). Quantitatively, the peak compressive stress remained highly stable, changing from 19.95 kPa in the 1st cycle to 19.82 kPa in the 5000th cycle, corresponding to 99.34% stress retention. The energy dissipation ratio remained low, increasing slightly from 5.21% to 6.27% for selected cycles (Figure 6b‐x), with full deformation recovery after 5000 cycles of testing. The near‐perfect overlap of the stress–strain curves at the 1st and 5000th cycles demonstrates excellent cyclic durability, elastic recovery, and structural robustness under prolonged cyclic deformation, in contrast to other soft hydrogels with notable hysteresis loop area over short‐term cyclic compression [56, 57, 58, 59]. This indicates insignificant chain scission, crosslink failure, or permanent network rearrangement of our hydrogels over long‐term cyclic loading. This result is particularly noteworthy given the high water content of our pre‐equilibrated hydrogel (> 90 wt.%), which typically renders hydrogel networks susceptible to failure by reducing fracture energy and fatigue threshold, according to the adapted Lake‐Thomas model for hydrogels [60, 61]. Free, non‐crosslinked chains, such as the linear PAAm formed during dark prepolymerization, could in principle promote creep or interfacial slippage under repeated loading, as they interact with the network only through non‐covalent interactions such as entanglements and dynamic hydrogen bonds [62, 63, 64]. No such effect was observed here: these chains did not compromise the cyclic durability of the gels. Whether they are fully retained in the network after equilibration in water was not determined.

## 3 Conclusion

This work demonstrates that dark redox prepolymerization is a simple and robust strategy for transforming inherently low‐viscosity aqueous resins into printable formulations for Xolography. This approach offers clear advantages for fabricating soft objects, complex support‐free structures, and multi‐materials. By controlling polymerization time to limit monomer conversion (< 30%), precursor resin viscosity was tuned on demand by more than four orders of magnitude (from 4.37 mPa s to beyond 90 000 mPa s) without altering the base resin chemistry. The unreacted monomer remained fully available for subsequent photopolymerization. Systematic printability mapping revealed that increasing viscosity (H2–H5) reduced the critical energy dose by up to 40% and suppressed gravity‐induced sedimentation. While the highest‐viscosity formulation (H6) successfully stabilized suspended rigid components, it required vigorous washing that damaged less‐crosslinked prints and thereby narrowed its printable light‐dose window. This trade‐off, i.e., maintaining sufficient viscosity for contactless fabrication without impeding part isolation, identified H5 (11037 ± 412 mPa s) as the optimal operating point for single‐material hydrogel printing, whereas H6 is preferred where sedimentation of embedded components dominates, as in multi‐material overprinting.

To further demonstrate the versatility of this prepolymerization strategy for contactless VAM, we also investigated a solvent‐free bulk resin system. Tuning the oxidizer concentration allowed viscous precursors to be prepared within 35 min at room temperature, increasing the resin viscosity from 4.12 to 1535 mPa s. This in situ thickening effectively converted an unprintable base formulation into a printable Xolography resin, which was validated by printing a high‐resolution (80 µm) object. Moreover, the successful fabrication of an interlocking waterwheel underscores the exceptional geometric versatility enabled by this contactless VAM strategy.

The approach was further validated through multi‐material Xolography, where sequential overprinting of rigid components and prepolymerized hydrogel resins (H6) produced a mechanically functional articular joint model with two mechanically isolated bone components embedded in a continuous soft tissue matrix. Cyclic compression testing confirmed highly reversible deformation analogous to the reversible coupling of an articular joint, demonstrating that the rigid–soft interface remained intact under repeated loading. An extended cyclic compression test demonstrated excellent elastic recovery and cyclic durability.

For biomedical applications, the APS/TEOA redox initiation system may raise potential biocompatibility concerns due to the presence of residual persulfates and tertiary amines. In the present study, however, the APS concentration was extremely low (0.0006 wt.%), corresponding to at most 6 ppm of residual persulfate; moreover, the negligible change in monomer conversion after one day of storage (Table S3) and over 14 days for the H6 precursor (Figure S12c‐ii) indicates that any residual APS does not initiate further polymerization. In addition, previous studies have reported that TEOA exhibits minimal cytotoxicity at concentrations up to 2.5 wt.% [16]. Although the TEOA concentration used in this work was higher (5 wt.%), its content could potentially be reduced through further formulation optimization. Alternatively, 2‐bis(2‐hydroxyethyl)amino‐2‐(hydroxymethyl)‐1,3‐propanediol (BIS‐TRIS) has emerged as a promising co‐initiator for aqueous photopolymerization systems, demonstrating excellent biocompatibility even at relatively high concentrations (800 × 10^−3^ M) [29]. These approaches may facilitate the future development of biocompatible resin systems for biomedical applications.

There are several limitations regarding the resin systems and polymerization chemistries compatible with the proposed in situ prepolymerization strategy. First, this method is not applicable to formulations composed exclusively of multifunctional crosslinkers, as premature gelation is likely to occur before a sufficiently viscous, yet processable precursor can be obtained. Second, the approach is not readily compatible with reversible‐deactivation radical polymerization (RDRP) techniques, such as reversible addition–fragmentation chain‐transfer (RAFT) polymerization. The strategy relies on the rapid formation of high‐molecular‐weight polymer chains at low monomer conversion, which is intrinsic to conventional free‐radical polymerization: a small number of long chains generated in the first minutes raises the viscosity by orders of magnitude while leaving the great majority of the monomer available for subsequent photopolymerization. In RDRP systems, by contrast, molecular weight increases progressively with monomer conversion, so the low conversions required to preserve printable monomer yield only short chains and negligible thickening, while the conversions needed to reach a printable viscosity would consume the monomer required for printing. The proposed strategy is therefore best suited to conventional free‐radical systems containing at least one monofunctional constituent, where viscosity can be raised substantially at low conversion while precursor processability is retained.

## Author Contributions

**Xichuan Li**: investigation, validation, formal analysis, data curation, Writing – original draft, Writing – review and editing. **Marcus Reuter**: writing – original draft, writing – review and editing, formal analysis, data curation. **Linguangze Zhuo**: investigation, validation, formal analysis, data curation, writing – original draft, writing – review and editing. **Xueheng Dai**: investigation, validation, writing – review and editing, formal analysis, data curation. **Camille Tribout**: validation, writing – review and editing, formal analysis, methodology. **Nathaniel Corrigan**: investigation, writing – review and editing, project administration, formal analysis, data curation, supervision, methodology. **Jin Zhang**: supervision, resources, project administration, writing – review and editing, validation, methodology. **Cyrille Boyer**: supervision, conceptualization, writing – original draft, funding acquisition, writing – review and editing, project administration, resources.

## Conflicts of Interest

Marcus Reuter is an employee of xolo GmbH. The remaining authors declare no conflicts of interest.

## Supporting information




**Supporting File 1**: advs78283‐sup‐0001‐SuppMat.docx.

**Supporting File 2**: advs78283‐sup‐0002‐VideoS1.mp4.

**Supporting File 3**: advs78283‐sup‐0003‐VideoS2.mp4.

## Data Availability

The data that support the findings of this study are available from the corresponding author upon reasonable request.
