# Supplementary material for: Prepolymerization Enables On‐Demand Viscosity Tuning for Xolography of Soft Hydrogels
Source: Adv Sci (Weinh). 2026 Oct 26:e78283. Online ahead of print. doi: 10.1002/advs.78283 (PMC13710707; doi:10.1002/advs.78283)
Supplement: Supplementary file 1 [file ADVS-9999-e78283-s001.docx]

Supporting Information

**Prepolymerization Enables On-Demand Viscosity Tuning for Xolography of Soft Hydrogels**

Xichuan Li,^1^ Marcus Reuter,^2^ Linguangze Zhuo,^3^ Xueheng Dai,^1^ Camille Tribout,^1^ Nathaniel Corrigan,^4^ Jin Zhang,^3*^ and Cyrille Boyer^1*^

^1^Cluster for Advanced Macromolecular Design, School of Chemical Engineering, University of New South Wales, Sydney, NSW 2052, Australia

^2^xolo GmbH, Volmerstraße 9B, 12489 Berlin, Germany

^3^School of Mechanical and Manufacturing Engineering, University of New South Wales, Sydney, NSW 2052, Australia

^4^Centre for Advanced Manufacturing Technology (CfAMT), School of Engineering, Western Sydney University, Sydney, NSW 2747, Australia

*Email: [jin.zhang6@unsw.edu.au](mailto:jin.zhang6@unsw.edu.au), [cboyer@unsw.edu.au](mailto:cboyer@unsw.edu.au)

**Materials**

All chemicals were used as received unless stated otherwise. Acrylamide (AAm, ≥99%), *N,N’*-methylenebisacrylamide (MBA, ≥98%), ammonium persulfate (APS, ≥98%), benzoyl peroxide (BPO, 25% water as stabilizer)**,** trimethylolpropane triacrylate (TMPTA, ≥ 60%) and pentaerythritol tetraacrylate (PETA, tetraacrylate by GC 60.0-90.0%, triacrylate by GC 10.0-40.0%) were purchased from Sigma-Aldrich. *N,N*-diethylacrylamide (DEAm, 98.0%) and triethanolamine (TEOA, 98%) were acquired from Chem-Supply. Deionized water (DI water) was prepared by a water purification device (ARIUM PRO, 18.2 MΩ cm). Dual-color photoinitiators (DCPI-6001, 650 g mol^-1^; DCPI-3001, 424 g mol^-1^) were provided by xolo GmbH.  Lithium phenyl-2,4,6-trimethylbenzoylphosphinate (LAP, ≥97%) was obtained from Ambeed. Deuterium oxide (D_2_O, 99.9%) was purchased from Cambridge Isotope Laboratories.

**Methods**

**Prepolymerization Protocol and Resin Preparation**

The hydrogel formulation comprised acrylamide (AAm, 45 wt.%, 1 equiv.) as monomer, *N,N*-diethylacrylamide (DEAm, 2.5 wt.%, 0.031 equiv.) as comonomer, *N,N'*-methylenebisacrylamide (MBA, 0.5 wt.%, 0.0051 equiv.) as crosslinker, triethanolamine (TEOA, 5 wt.%, 0.053 equiv.) as reducing agent, ammonium persulfate (APS, 0.0006 wt.%, 0.0000042 equiv.) as oxidizer, dual-color photoinitiator (DCPI-6001, 0.05 wt.%, 0.00012 equiv.) as photoinitiator, and deionized water (DI water, 46.95 wt.%, 4.12 equiv.) as solvent. Here, DEAm facilitated dissolution of DCPI-6001 in the aqueous resin, while TEOA served dual roles as both accelerator for redox prepolymerization and coinitiator for Xolography. A typical protocol for preparing 16000 mg of hydrogel resin is as follows.

For prepolymerization, AAm (7200 mg) and DI water (4605.3 mg) were added to a glass vial (21 mL) containing a PTFE stir bar (20 × 6 mm). The mixture was sonicated at 40 °C until fully dissolved, then cooled to room temperature (24 °C). TEOA (800 mg) was added, and the vial was sealed with a rubber stopper and vortexed to mix. The solution was purged with nitrogen for 5 min. Separately, a fresh APS solution (0.03 wt.%) was prepared, and 320 mg (APS: 0.096 mg; DI water: 319.904 mg) was injected into the sealed vial, followed by an additional 0.5 min of degassing. All needles were then removed and magnetic stirring commenced to begin prepolymerization at 24 °C. The start of prepolymerization (t = 0 s) was defined as the point of APS addition. To obtain precursors of varying viscosity, prepolymerization was quenched at targeted time points by removing the rubber stopper, exposing the mixture to air, and immersing the vial in an ice bath. Simultaneously, the precursor was stirred manually for 5 min to accelerate oxygen diffusion and cooling. Any precipitates formed during the ice bath were redissolved on rotating rollers at room temperature. The maximum prepolymerization time for the most viscous precursor was under 20 min (1110 s), yielding a total preparation time—including degassing and quenching—of under 30 min.

For resin preparation, a 3 wt.% MBA stock solution (MBA: 80 mg; DI water: 2586.7 mg) was added to the prepolymerized precursor. High-viscosity groups were mixed using rotating rollers, while all others used magnetic stirring. DCPI-6001 (8 mg) was dissolved in DEAm (400 mg) and added to the mixture, followed by further mixing for homogenization. The resin was transferred to polystyrene cuvettes (Sigma-Aldrich), sealed with parafilm, and centrifuged at 6000 rpm for 1 min at 24 °C to remove entrapped air bubbles. All samples were stored in the dark prior to printing.

The solvent-free formulation consisted of diethylacrylamide (DEAm, 71.20 wt.%, 1 equiv.) as monomer, trimethylolpropane triacrylate (TMPTA, 23.73 wt.%, 0.14 equiv.) as crosslinker, triethanolamine (TEOA, 5.00 wt.%, 0.060 equiv.) as reducing agent, benzoyl peroxide (BPO, 0.015 wt.%, 0.00011 equiv.) as oxidizer, and dual-color photoinitiator (DCPI-3001, 0.05 wt.%, 0.00021 equiv.) as photoinitiator. Here, TEOA served dual roles as both accelerator for redox prepolymerization and coinitiator for Xolography.

A typical protocol for preparing viscous precursors and resins (16000 mg) is as follows: TEOA (800.00 mg) was added to liquid-state DEAm (10522.60 mg), followed by nitrogen purging for 5 min. Separately, a fresh BPO solution (0.5 wt.%), consisting of BPO (2.4 mg) and DEAm (477.60 mg), was injected into the monomer solution after 5-min degassing, followed by an additional 0.5-min purging. The start of prepolymerization (t = 0 s) was defined as the point of BPO addition. Quenching was conducted at the targeted time point by removing the rubber stopper, exposing the mixture to air, and immersing the vial in an ice bath. Simultaneously, the precursor was stirred manually for 5 min to accelerate oxygen diffusion and cooling. Precursors were put on rotating rollers at room temperature to further consume residual BPO with introduced oxygen. To prepare resins for printing, TMPTA (3797.40 mg) was added to prepolymerized precursors, obtaining a homogeneous mixture via rigorous vortexing. Subsequently, a DCPI solution comprising DCPI-3001 powder (8 mg) and DEAm (392 mg) was added to the mixture, with further mixing on rotating rollers. The resin was dispensed into UV-transparent cuvettes, sealed with parafilm, and centrifuged at 6000 rpm for 1 min at 24 °C to remove entrapped air bubbles. All samples were stored in the dark prior to printing.

For multi-material fabrication, another solvent-free formulation, consisting of 94.99 wt.% PETA, 5 wt.% TEOA and 0.01 wt.% DCPI, was designed to fabricate rigid components.

**Conventional thickening protocol using purified polymers**

As a benchmark comparison against our *in situ* strategy, a control resin was prepared by adding an externally pre-synthesized and purified polymer (PAAm) into the base formulation. To prepare polyacrylamide (PAAm) as an external thickener, the same prepolymerization protocol used for the highest-viscosity formulation (H6) was employed, rather than using commercial PAAm, to ensure a like-for-like comparison with the in situ approach. The resulting polymer was subsequently purified by dialysis against deionized water using a 3500 Da molecular weight cut-off (MWCO) membrane for 3 days, followed by lyophilization to remove residual water and obtain the PAAm thickener. ^1^H NMR spectroscopy was used to confirm that residual monomer was completely removed.

The control resin formulation was designed based on the composition of the highest-viscosity formulation, H6, consisting of AAm (39.88 wt.%), PAAm (15.82 wt.%), TEOA (6.19 wt.%), and DI water (38.11 wt.%). Initially, all components except PAAm were mixed to form a homogeneous base formulation. Subsequently, the external thickener (PAAm) was added in three equal portions (5.27 wt.% each); however, the second portion could not be fully dissolved, and poor miscibility was observed (**Figure S12bii, Supporting Information**). To facilitate dissolution of the polymer additive and improve resin homogeneity, heating at 40 °C and roller mixing at room temperature were alternated throughout the preparation process.

**Xolography**

The Xube^2^ printer employs a two-light projection system comprising a 405-nm laser light sheet (2 × 350 mW diode lasers) and a 3840 × 2160 pixel (UHD) digital light projector with a mercury lamp emitting in the visible-light range (520 & 613 nm, referred to as red light). The maximum build volume is 10 × 17.8 × 10 mm (x, y, z). Digital models were designed in Fusion 360, exported as .stl files, and imported into the Xube^2^ software for position and size adjustment. Print parameters, including position, speed, and 405-nm light-sheet light dose, were set prior to printing. Single-material prints followed a continuous process, while multi-material prints used sequential fabrication. The irradiated light dose as a function of light-sheet intensity, speed, and optical path length is given in **Equation S1 (Supporting Information)**.

Light dose = $3\cdot\frac{\mathrm{Intensity}}{\mathrm{Speed}\cdot l}$	**(****Equation S1)**

where intensity (mW mm^-2^) is the power of the 405-nm laser light sheet per unit area of the resin vat; speed (mm min^-1^) is the translation rate of the print vat; *l* (10 mm) is the optical path length of the laser light sheet through the resin; and light dose (mJ mm^-3^) is the total energy delivered to per unit volume of resin during printing. The light-sheet thickness is assumed to be 50 µm.^1^

After printing, hydrogels were isolated by diluting and dissolving the uncured resin with DI water, followed by removal via pipetting. The cuvette was then refilled and rinsed repeatedly with DI water until no residual resin remained. The printed object was subsequently post-cured in a LAP solution (0.1 wt.%) for 5 min under UV light (*λ* = 365 nm). All hydrogels were stored in DI water before any further testing.

Solvent-free prints were washed with diethyl ether before a 5-min post-curing under UV light (*λ* = 365 nm).

For rigid materials printed using PETA, diethyl ether was adopted to clean green parts, before being isolated and irradiated with 5-min UV light (*λ* = 365 nm).

**Nuclear Magnetic Resonance (NMR)**

All ^1^H NMR spectra were recorded on a Bruker Avance III 400 MHz spectrometer with 16 scans per sample, with chemical shifts reported in ppm (*δ*) relative to deuterium oxide (D_2_O).

**Size-Exclusion Chromatography (SEC)**

For hydrogel formulation, polymer molecular weight and dispersity were measured using an aqueous size-exclusion chromatography system (Prominence, Shimadzu) equipped with two PL aquagel-OH MIXED columns (8 µm; Agilent) and a differential refractive index detector (RID-10A). The mobile phase was 70 mM phosphate buffer (pH 9) with 0.2 M NaNO_3_ and 20 v/v% MeOH running at a flow rate of 1 mL min^-1^. Molecular weight calibration was performed using refractive index detection and near-monodisperse poly(ethylene oxide) standards (PL2080-0201, Agilent).

To characterize solvent-free prepolymers, the SEC system consisted of *N,N*-Dimethylacetamide (DMAc) eluent (containing 0.03% w/v LiBr and 0.05% w/v 2,6-dibutyl-4-methylphenol (BHT)) at 50 °C (flow rate of 1 mL min^-1^) with a Shimadzu modular system comprising a SIL-10AD auto-injector, a Polymer Laboratories 5.0 μm bead-size guard column (50 × 7.5 mm) followed by three linear PL (Styragel) columns (10^5^ Å, 10^4^ Å, 10^3^ Å) and an RID-10A differential refractive index (RI) detector. The equipment was calibrated based on commercial poly(methyl methacrylate) (PMMA) standards with molecular weight of 200–10^6^ g mol^-1^.

**Ultraviolet-Visible Light (UV–Vis) Spectroscopy**

All UV–Vis spectra were recorded on a Varian Cary 300 spectrophotometer over 300–800 nm at 600 nm min^‑1^, with the instrument zeroed at 800 nm using a quartz cuvette. To determine the molar extinction coefficient of DCPI, a series of DCPI/DEAm solutions were prepared at concentrations ranging from 0.00046 to 0.00185 mol L^-1^, yielding absorbance values of 0.2–1.0. Nine data points were measured at 405 nm (corresponding to the laser light-sheet wavelength) to construct a calibration curve of absorbance versus concentration. The molar extinction coefficient was calculated using **Equation S2 (Supporting Information)**.

*A* = *ε*‧*c*‧*l*	**(Equation S2)**

where *A* is the absorbance, *ε* (L mol^-1^ cm^-1^) is the molar extinction coefficient, *c* (mol L^-1^) is the concentration of DCPI, and *l* (cm) is the optical path length through the resin.

**Light Intensity Distribution**

On the Xube^2^, the 405-nm laser is split into two beams of equal intensity (0.5 *I*_0_, where *I*_0_ is the incident intensity before resin penetration), irradiating the print vat from opposite sides. UV–Vis spectra confirm that DCPI (*ε* = 544 L mol^-1^ cm^-1^) is the dominant absorber of violet light among all resin components, governing the 405-nm intensity distribution within the cuvette during printing (**Figure S2, Supporting Information)**. The intensity profile was calculated using the Beer–Lambert law (**Equation S2, Supporting Information**).

**Viscosity Measurements**

Viscosity was measured using a Brookfield DV3T viscometer equipped with a CPA-52Z (prepolymerized precursors) or CPA-42Z (unprepolymerized monomer solutions) spindle and TC-150SD temperature controller at 25 ± 0.2 °C. Data points were recorded every 10 s over 5–30 min until steady-state values were reached. Measurements were considered valid only when torque remained within the recommended range (10–100%).

**Optical Microscopy**

Sharp features of printed hydrogels were imaged on an AmScope microscope using a 10× objective and 10× eyepiece. Star-shaped hydrogels were stained with Rhodamine B (1 mg mL^-1^) for contrast.

**Scanning electron microscopy (SEM)**

All samples were prepared on a stub with conductive tape and coated with a 30 nm platinum layer using a Leica ACE600 sputter coater. SEM images were acquired using a field-emission NanoSEM 230 instrument operated at a 3 kV accelerating voltage and a secondary electron detector.

**Mechanical testing**

The mechanical properties of the hydrogels were evaluated by compression testing using a Mark–10 ESM303 mechanical test stand equipped with parallel compression plates. A 1 kN force gauge was used for rigid samples, while a 25 N force gauge was used for hydrogels. Cylindrical hydrogel specimens were printed and equilibrated in deionized water to reach a fully hydrated state. Excess surface water was gently removed using clean tissue before monotonic compression measurements. For hysteresis and long-term cyclic compression tests, the samples were fully immersed in deionized water throughout testing to simulate the aqueous environment.

Monotonic compression tests were performed at a loading rate of 1 mm min^-1^ until the force gauge reached the upper limit (rigid PETA samples) or fracture occurred (hydrogels). Cyclic compression stability tests were performed at 5 mm min^-1^ for 100 cycles, and 10 mm min^-1^ for 5000 cycles.

The engineering compressive strain was calculated as **Equation S3 (Supporting Information)**:

$Engineering strain \left( \varepsilon\right)=\frac{{(h}_{0}-h)}{h_{0}}$	(**Equation S3**)

where $h_{0}$ and $h$ are the initial and instantaneous sample heights, respectively. The engineering compressive stress was calculated as **Equation S4 (Supporting Information)**:

$Engineering stress \left( \sigma\right)=\frac{F}{A_{0}}$	(**Equation S4**)

where $F$ is the recorded compressive force and $A_{0}$ is the initial cross-sectional area of the cylindrical hydrogel sample.

The compressive modulus was determined from the slope of the linear elastic region of the stress–strain curve, typically within 10–20% strain, using **Equation S5 (Supporting Information)**:

$E_{c}=\frac{\Delta\sigma}{\Delta\varepsilon}$	(**Equation S5**)

where *E_c_* is the compressive modulus, $\Delta\sigma$ is the change in compressive stress, and $\Delta\varepsilon$ is the corresponding change in strain within the elastic region.

**Figure S1. Formulation optimization and Xolography using one-step protocol.** (**a**) Effect of crosslinker concentration (MBA) on precursor processability after prepolymerization. Formulations containing (i) 0.5, (ii) 0.1 and (iii) 0.06 wt.% MBA could not be prepolymerized into a viscous yet flowable resin, as premature gelation occurred before sufficient viscosity development was achieved; the photograph shows the gelled 0.06 wt.% MBA formulation. Only (iv) 0.04 wt.% MBA yielded a processable precursor. (**b**) Photographs of printed structures obtained at different light doses while maintaining a constant printing speed of 1 mm min^-1^. A precursor containing 0.04 wt.% MBA was prepolymerized to produce a viscous yet flowable resin suitable for printing. Scale bars are 5 mm.

**Table S1.** Hydrogel formulation for prepolymerization-enabled Xolography.

|  | **AAm** | **DEAm** | **MBA** | **DI water** | **TEOA** | **DCPI** | **APS** |
| --- | --- | --- | --- | --- | --- | --- | --- |
| **Mass fraction (wt.%)** | 45 | 2.5 | 0.5 | 46.95 | 5 | 0.05 | 0.0006 |
| **Molar ratio** | 1 | 0.031 | 0.0051 | 4.12 | 0.053 | 0.00012 | 0.0000042 |

**Notes**: AAm, MBA, and DI water serve as monomer, crosslinker, and solvent, respectively. A small amount of DEAm is included as a comonomer to facilitate DCPI dissolution in the aqueous mixture. APS acts as an oxidizer as the redox pair for dark prepolymerization. TEOA serves a dual role as both reducing agent for dark prepolymerization and coinitiator for Xolography.

**Figure S2. UV–Vis spectra of resin components with relevant calculations.** (**a**) Light absorption of each component in the hydrogel formulation, with the dashed line at 405 nm indicating the violet light-sheet wavelength used in Xolography. (**b)** Calibration curve of absorbance versus DCPI concentration, with 0.05 wt.% DCPI adopted in this work. (**c**) Molar extinction coefficient (*ε*) of DCPI as a function of wavelength, calculated using **Equation S2 (Supporting Information)**; *ε* = 544 L mol^-1^ cm^-1^ at 405 nm. (**d**) Light intensity distribution profiles of hydrogel resins with varying DCPI concentrations, referring to **Light Intensity Distribution (Supporting Information).**

Maximum light attenuation (*I*_a_) was 0.5, 10.6 and 82.2% at DCPI loadings of 0.01, 0.05 and 0.25 wt.%, calculated using **Equation S6 (Supporting Information):**

$I_{a}=\frac{I_{1.0}-I_{0.5}}{I_{1.0}}\cdot100\%$	**(Equation S6)**

where *I*_0.5_ and *I*_1.0_ denote the 405-nm light intensity at optical path lengths of 0.5 cm and 1.0 cm within the print vat (cuvette), respectively.

**Table S2**. Effect of APS concentration on quenching efficiency.

| **Formulation** | **APS concentration (wt.%)^a^** | **Prepolymerization time (s)^b^** | **Viscosity (mPa s)** | **Post-quenching**  **polymerization^c^** |
| --- | --- | --- | --- | --- |
| **1** | 0.0016 | 283 | N/A^d^ | Yes |
| **2** | 0.0008 | 392 | 220 | No |
| **3** | 0.0006 | 590 | 145.7 | No |
| **4** | 0.0004 | 892 | 135.5 | No |

^a^-Prepolymerization formulations were designed based on **Table S1** (**Supporting Information**), containing only AAm, DI water, and TEOA mixed with varying APS concentrations. ^b^-Prepolymerization was quenched by monitoring stir bar movement to obtain comparable viscosity values among testing groups. ^c^-High APS concentrations resulted in the presence of significant amount of residual APS, which continued to decompose after quenching, followed by exothermic heating and gel formation. ^d^-Not measurable due to solidification after quenching.

**Table S3.** Monomer conversion tracking after prepolymerization using 0.0006 wt.% APS.

| **ID** | **Prepolymerization time (s)** | **Monomer conversion (%)** | |
| --- | --- | --- | --- |
|  |  | **0 h after**  **Prepolymerization** | **24 h after prepolymerization** |
| **H1** | 0 | 0 | 0 |
| **H2** | 590 | 5.85 | 5.93 |
| **H3** | 815 | 15.03 | 15.10 |
| **H4** | 950 | 20.73 | 20.87 |
| **H5** | 1070 | 24.45 | 24.72 |
| **H6** | 1110 | 28.42 | 28.83 |

**Notes**: All precursors were prepared using the formulation in **Table S1** (**Supporting Information**) and quenched at varying reaction times to obtain a range of viscosities for subsequent experiments. Monomer conversion was determined by ^1^H NMR. Precursors were stored at room temperature (24 °C) for 24 h.

**Table S4.** Characterization of prepolymerized precursors and resins as a function of prepolymerization time (individual replicates; mean ± SD (STDEV.P) values are given in Table 1).

| **ID** | **Prepolymerization time (s)** | **Precursor** | | | |  | **Resin^d^** |
| --- | --- | --- | --- | --- | --- | --- | --- |
|  |  | **Conversion^a^ (%)** | ***M*_n_^b^**  **(kg mol^-1^)** | ***Ð*^b^** | **Viscosity^c^**  **(mPa s)** |  | **Viscosity^c^**  **(mPa s)** |
| **H1** | 0 | 0 | N/A | N/A | 4.37 |  | 3.10 |
| **H2** | 590 | 5.81 | 229.7 | 2.18 | 138.1 |  | 71.44 |
|  |  | 5.85 | 220.1 | 2.28 | 142.8 |  | 73.86 |
|  |  | 6.03 | 221.8 | 2.24 | 156.3 |  | 74.79 |
| **H3** | 815 | 14.76 | 203.1 | 2.20 | 4955 |  | 1496 |
|  |  | 15.03 | 195.9 | 2.25 | 5110 |  | 1501 |
|  |  | 15.66 | 205.2 | 2.20 | 5451 |  | 1619 |
| **H4** | 950 | 20.54 | 195.8 | 2.17 | 19950 |  | 4930 |
|  |  | 20.73 | 195.9 | 2.19 | 21580 |  | 5423 |
|  |  | 20.39 | 193.2 | 2.23 | 22600 |  | 5386 |
| **H5** | 1070 | 24.51 | 189.9 | 2.18 | 50320 |  | 11590 |
|  |  | 24.45 | 194.5 | 2.16 | 51630 |  | 10600 |
|  |  | 24.60 | 190.1 | 2.16 | 53210 |  | 10920 |
| **H6** | 1110 | 27.90 | 146.8 | 2.08 | 87810 |  | 16860 |
|  |  | 28.42 | 163.1 | 2.12 | 96000 |  | 18650 |
|  |  | 28.72 | 174.3 | 2.16 | 99530 |  | 20280 |

^a^-Monomer conversion was determined by ^¹^H NMR from the integral ratio of CH₂ and CH protons of monomers and prepolymers, with D_2_O as solvent (**Figure S3a, Supporting Information**). ^b^-Number-average molecular weight (*M*_n_) and polymer molecular weight distribution (*Ð*) were characterized by aqueous SEC (phosphate buffer (pH = 9) as the eluent, poly(ethylene oxide) as the standards). ^c^-Viscosity was measured at 25 ± 0.2 °C using a viscometer equipped with a CPA-52Z spindle for H2–H6 and a CPA-42Z spindle for H1. ^d^-Resins were prepared by adding crosslinker and DCPI to the prepolymerized precursors. The viscosity of H1-H6 precursors was measured at 384, 40, 3, 0.4, 0.2, 0.1 s^-1^, respectively. The viscosity of H1-H6 resins was measured at 384, 100, 10, 2.0, 1, 0.4 s^-1^, respectively.

**Figure S3. Characterization of prepolymerized precursors.** (**a**) ^1^H NMR spectra of precursors obtained at varying reaction times: t = 0 s (H1), 590 s (H2), 815 s (H3), 950 s (H4), 1070 s (H5), and 1110 s (H6). Monomer conversion was calculated from AAm (*e, f, g*) and PAAm (*a, b*) signals. (**b**) SEC traces of prepolymers at varying reaction times, using phosphate buffer (pH 9) as eluent and poly(ethylene oxide) as calibration standards. (**c**) ln([M]_0_/[M]*_t_*) and viscosity as a function of reaction time (*t*); data are presented as mean ± SD (*n* = 3).

**Table S5.** Prepolymerized resin formulations for Xolography.

| **ID** | **AAm (wt.%)** | **PAAm (wt.%)** | **DEAm (wt.%)** | **MBA (wt.%)** | **DI water (wt.%)** | **TEOA (wt.%)** | **DCPI-6001 (wt.%)** | **APS**  **(wt.%)** |
| --- | --- | --- | --- | --- | --- | --- | --- | --- |
| **H1** | 45 | 0 | 2.5 | 0.5 | 46.95 | 5 | 0.05 | Depleted |
| **H2** | 42.345 | 2.655 |  |  |  |  |  |  |
| **H3** | 38.182 | 6.818 |  |  |  |  |  |  |
| **H4** | 35.752 | 9.248 |  |  |  |  |  |  |
| **H5** | 33.966 | 11.034 |  |  |  |  |  |  |
| **H6** | 32.242 | 12.758 |  |  |  |  |  |  |

**Notes**: Mass fractions of AAm and PAAm were calculated from monomer conversion data in **Table 1**. Consumption of TEOA during prepolymerization was neglected given the minuscule APS concentration used (0.0006 wt.%).

**Figure S4.** Light-dose printability windows for hydrogels fabricated using the base resins **(**H1, 3.10 mPa s) without prepolymerization. Speed: 1 mm min^-1^; Digital model: dumbbell (12 × 4 × 1 mm). Scale bars: 5 mm.

**Figure S5.** Light-dose printability windows for hydrogels fabricated using prepolymerized resins **(**H2, 73.36 ± 1.41 mPa s). Green denotes well-defined structures. Speed: 1 mm min^-1^; Digital model: dumbbell (12 × 4 × 1 mm). Scale bars: 5 mm.

**Figure S6.** Light-dose printability windows for hydrogels fabricated using prepolymerized resins **(**H3, 1539 ± 57 mPa s). Green denotes well-defined structures. Speed: 1 mm min^-1^; Digital model: dumbbell (12 × 4 × 1 mm). Scale bars: 5 mm.

**Figure S7.** Light-dose printability windows for hydrogels fabricated using prepolymerized resins **(**H4, 5246 ± 224 mPa s). Green denotes well-defined structures. Speed: 1 mm min^-1^; Digital model: dumbbell (12 × 4 × 1 mm). Scale bars: 5 mm.

**Figure S8.** Light-dose printability windows for hydrogels fabricated using prepolymerized resins **(**H5, 11037 ± 412 mPa s). Green denotes well-defined structures. Speed: 1 mm min^-1^; Digital model: dumbbell (12 × 4 × 1 mm). Scale bars: 5 mm.

**Figure S9.** Light-dose printability windows for hydrogels fabricated using prepolymerized resins **(**H6, 18597 ± 1397 mPa s). Green denotes well-defined structures. Speed: 1 mm min^-1^; Digital model: dumbbell (12 × 4 × 1 mm). Scale bars: 5 mm.

**Figure S10.** Photographs of printed hydrogels at their best curing state prior to isolation. Scale bars: 5 mm.

**Table S6.** Quantitative calculations of sedimentation.

| **Parameters** | **H1** | | **H2** | | **H3** | | **H4** | | **H5** | | **H6** | |
| --- | --- | --- | --- | --- | --- | --- | --- | --- | --- | --- | --- | --- |
|  | **Disk** | **Star** | **Disk** | **Star** | **Disk** | **Star** | **Disk** | **Star** | **Disk** | **Star** | **Disk** | **Star** |
| **Δ*h* (mm)** | N/A | N/A | N/A | N/A | 0.539 | 2.476 | 0.096 | 1.263 | 0.023 | 0.337 | 0.011 | 0.040 |
| ***h* (mm)** | 17.8 | | | | | | | | | | | |
| **Sedimentation number (*N*_s_)** | N/A | N/A | N/A | N/A | 0.030 | 0.139 | 0.005 | 0.071 | 0.001 | 0.019 | 0.001 | 0.002 |

**Notes**: Sedimentation was calculated based on **Equation S7 (Supporting Information)**:

$Ns= \frac{t_{\mathrm{print}}}{t_{s}}$	(**Equation S7**)

where *N*_s_ is sedimentation number; *t*_print_ represents exposure time during Xolography; *t*_s_ indicates characteristic sedimentation time. Specifically, *t*_s_ = *h*/*v*_s_, where *h* symbolizes height of irradiated volume; *v*_s_ is the settling velocity, calculated using **Equation S8 (Supporting Information):**

$v_{s}= \frac{\Delta h}{t_{\mathrm{print}}}$	(**Equation S8**)

Here, *Δh* is the settling displacement of the first printed layer relative to the last printed layer over the fabrication process. Then sedimentation number is calculated based on **Equation S9 (Supporting Information)**:

$Ns= \frac{t_{\mathrm{print}}}{t_{s}}= \frac{t_{\mathrm{print}}}{\frac{h}{v_{s}}}= \frac{t_{\mathrm{print}}\times v_{s}}{h}= \frac{t_{\mathrm{print}}\times\Delta h}{h \times t_{\mathrm{print}}}= \frac{\Delta h}{h}$	(**Equation S9**)

**Figure S11.** Sharpness characterization of printed stars using prepolymerized resins, with feature width indicated by white lines around the tip. Feature widths measured by ImageJ were 39, 38, 38, 38, and 30 µm for H2–H6, respectively. Rhodamine B (1 mg mL^-1^) was added to enhance contrast under microscopy. H1 (no prepolymerization) was not printable by Xolography. Scale bars: 300 µm.

**Table S7.** Quantitative resolution analysis of printed hydrogels.

| **ID** | **Size of printed samples (mm)** | | | | | | | | | |
| --- | --- | --- | --- | --- | --- | --- | --- | --- | --- | --- |
|  | **Dumbbell** | | | **Disk** | | | | **Star** | | |
|  | **Length** | **Height** | **Thickness** | **Diameter** | **Thickness** | **Hollow circle** | **Hollow triangle** | **Diameter** | **Thickness** | **Sharpness** |
| **Model** | 4.0 | 12.0 | 1.0 | 6.0 | 2.0 | 2.0 | 1.4 | 6.0 | 4.0 | 0 |
| **H1** | N/A | N/A | N/A | N/A | N/A | N/A | N/A | N/A | N/A | N/A |
| **H2** | 5.5 | 16.0 | 0.9 | 7.5 | 1.9 | 2.9 | 1.9 | 5.1 | 1.9 | 0.039 |
| **H3** | 5.5 | 16.4 | 0.9 | 7.4 | 2.5 | 2.6 | 1.5 | 6.6 | 4.3 | 0.038 |
| **H4** | 5.4 | 16.0 | 1.1 | 7.1 | 2.3 | 2.6 | 1.4 | 6.5 | 4.1 | 0.038 |
| **H5** | 5.6 | 16.1 | 1.1 | 7.5 | 2.3 | 2.8 | 1.7 | 6.5 | 4.4 | 0.038 |
| **H6** | 5.4 | 16.3 | 1.1 | 7.5 | 2.3 | 2.5 | 1.4 | 6.2 | 4.3 | 0.030 |
| **Deviation*** | 0.40 | 0.37 | 0.1 | 0.25 | 0.15 | 0.40 | 0.21 | 0.08 | 0.10 | N/A |

**Notes**: high-fidelity printouts were labeled with green color. *-Deviation was calculated using **Equation S10 (Supporting Information)**:

$D= \frac{l_{\max}-l_{\mathrm{model}}}{l_{\mathrm{model}}}$	(**Equation S10**)

where *l*_max_ (mm) is the largest measured size among well-cured materials (green color) for each model; *l*_model_ (mm) indicates the model size.

**Table S8.** Water content measurement of disk hydrogels.

| **Formulation** | **Wet mass (mg)** | **Dry mass (mg)** | **Water content (wt.%)** |
| --- | --- | --- | --- |
| **H1** | N.A. | N.A. | N.A. |
| **H2** | 88.40* | 3.44 | 96.11 |
| **H3** | 121.84 | 6.42 | 94.73 |
| **H4** | 135.80 | 9.74 | 92.83 |
| **H5** | 142.46 | 9.09 | 93.62 |
| **H6** | 129.98 | 6.97 | 94.64 |

***-**Low wet mass was obtained due to low viscosity-induced poor resolution. The equilibrium water content was calculated using **Equation S11 (Supporting Information)**:

$w= \frac{m_{W}-m_{D}}{m_{W}}\times100\%$	(**Equation S11**)

where *m*_W_ (mg) is mass of the fully equilibrated disk in DI water; *m*_D_ (mg) is mass of the freeze-dried disk.

**Figure S12. Preparation of viscous precursor using pre-synthesized polyacrylamide (PAAm).** (**a**) (**i**) ^1^H NMR spectra of polyacrylamide before and after purification. The monomer conversion was calculated to be 28.1 %; (**ii**) SEC trace of synthesized purified PAAm. (**b**) Comparison of in situ prepolymerization and the conventional thickening approach using an external polymer: (**i**) Composition, viscosity and preparation time of H6 precursor; (**ii**) Preparation of a viscous precursor using externally pre-synthesized PAAm. To facilitate homogeneous mixing of AAm and PAAm in the highly viscous solution, alternating heating at 40 °C and roller mixing at room temperature were employed. (**c**) (**i**) Light transmittance spectra of precursors prepared using the in situ prepolymerization strategy and the conventional PAAm-thickening approach, measured after 1 and 14 days of storage; (**ii**) Long-term stability of the H6 precursor, showing unchanged light transmittance and monomer conversion, and a viscosity increase of less than 7%, between 1 and 14 days of storage.

**Figure S13.** Chemical structures of the solvent-free formulation.

**Table S9.** Optimization of BPO concentration for solvent-free prepolymerization

| **ID^a^** | **BPO concentration (wt.%)** | **Post-quenching polymerization^b^** |
| --- | --- | --- |
| **1** | 0.050 | Yes |
| **2** | 0.025 | Yes |
| **3** | 0.015 | No |
| **4** | 0.010 | No^c^ |

**Notes**: ^a^-Prepolymerization formulations were designed based on **Table S10** (**Supporting Information**), containing DEAm and TEOA with varying BPO concentrations. ^b^-Viscosity of prepolymerized precursors at 0 and 12 h was measured to assess the occurrence of post-quenching polymerization. ^c^-At 0.010 wt.% BPO concentration, long reaction time (> 2 h) was required to prepare viscous resin (> 1000 mPa s).

**Table S10.** Resin composition for the solvent-free system

| **ID** | **wt.%** | | | | |
| --- | --- | --- | --- | --- | --- |
|  | **DEAm** | **TMPTA** | **TEOA** | **BPO** | **DCPI-3001** |
| **P1** | 71.20 | 23.73 | 5.00 | 0 | 0.05 |
| **P2** | 71.20 | 23.73 | 5.00 | 0.015 | 0.05 |

**Notes**: P1 is the base formulation without prepolymerization, whereas P2 represents the optimized formulation used to prepare viscous resins for high-fidelity print via the two-step protocol.

**Figure S14. Characterization of prepolymerized precursors.** (**a**) ^1^H NMR spectra of the precursor quenched at t = 35 min. Monomer conversion was calculated from DEAm (*a, b, d*) and broadened PDEAm (*j*) signals. (**b**) SEC trace of prepolymers quenched at t = 35 min (P2), using DMAc as eluent and poly(methyl methacrylate) as calibration standards. (**c**) Viscosity measurement of precursor and resin prepared using P2 formulation.

**Figure S15.** Compressive stress-strain curves of (**a**) rigid materials and (**b**) soft hydrogels. Data are reported as mean ± SD; *n* = 4.

**Figure S16.** Cyclic compression testing. (**a**) Stress-cycle behavior of a disk (H6) at a compression strain amplitude of 30% in aqueous conditions. (**b**) Hysteresis loop curves of the hydrogel at the beginning (the 1st) and end (the 100th) of cyclic compression testing.

**Supporting Information-Reference**

1.	Li X, Xiu Y, Lee K, Zhang J, Corrigan N, Boyer C. Xolography for Rapid Volumetric Production of Objects from the Nanoscopic to Macroscopic Length Scales. *Adv. Mater.* **37**, e2503245 (2025).
